# Simulated CO_2_ Fertilization Drives Progressive Phosphorus Limitation via Accelerated Organic Cycling in Amazonian Forests

**DOI:** 10.1111/gcb.71000

**Published:** 2026-07-20

**Authors:** Katrin Fleischer, Lin Yu, Lucia Fuchslueger, Carlos A. Quesada, Sönke Zaehle

**Affiliations:** ^1^ Systems Ecology, Faculty of Science Vrije Universiteit Amsterdam Amsterdam the Netherlands; ^2^ Biogeochemical Signals Department Max‐Planck‐Institute for Biogeochemistry Jena Germany; ^3^ Department of Earth System Sciences Hamburg University Hamburg Germany; ^4^ Centre of Microbiology and Environmental Systems Sciences University of Vienna Vienna Austria; ^5^ National Institute for Amazonian Research Manaus Brazil

**Keywords:** Amazonian tropical forests, CO_2_ fertilization, microbial modeling, organic phosphorus cycling, phosphorus mineralization, soil phosphorus

## Abstract

Tropical forest responses to increasing atmospheric CO_2_ (iCO_2_) depend on nutrient constraints, with phosphorus emerging as a central limiting factor. However, current global land models rarely include explicit representations of microbial‐driven phosphorus cycling, which is critical in highly weathered tropical soils. Here, we use the QUINCY land surface model coupled with the microbial‐explicit Jena Soil Model (JSM) to simulate coupled carbon–nutrient dynamics in Amazon forests along a natural gradient in soil phosphorus availability (40–312 g P m^−2^). The model reproduced observed patterns of increasing wood production with soil phosphorus and identified a threshold of ~80 g P m^−2^ in the topsoil below which plants experienced phosphorus limitation, shifting carbon allocation belowground and reducing foliar nitrogen : phosphorus ratios. Across all sites, microbial mineralization of organic phosphorus supplied the greatest share of soil solution phosphorus, with stronger reliance on organic phosphorus cycling in low‐phosphorus sites. The observed rise in atmospheric CO_2_ between 1901 and 2019 increased simulated plant and soil carbon stocks by 23.3% and 1.5%, respectively. However, forests on low‐phosphorus soils accumulated 40% less additional plant carbon under iCO_2_ than forests on high‐phosphorus sites. CO_2_ stimulated greater fine‐root carbon growth (+29.5%) and higher biochemical phosphorus mineralization (+24.5%) in low‐phosphorus sites. These responses increased reliance on organic phosphorus cycling and depleted mineral‐associated organic phosphorus pools. In contrast, phosphorus demand in high‐phosphorus forests was largely met by inorganic soil phosphorus pools. Across the gradient, phosphorus turnover increased and phosphorus was reallocated from slow‐turnover soil pools into living biomass, with the strongest shifts in low‐phosphorus sites. Together, these responses indicate emerging progressive phosphorus limitation under iCO_2_, characterized by increased belowground carbon allocation, greater reliance on organic phosphorus cycling, and increasing pressure on organic phosphorus pools through enhanced turnover that may not be sustained over time.

## Graphical abstract

Rising atmospheric CO_2_ increases phosphorus demand in Amazon forests. Using a microbial‐explicit land model in low and high phosphorus Amazonian forest sites, we show that low‐phosphorus forests increasingly rely on organic phosphorus cycling to sustain productivity. Increasing CO_2_ led to higher plant productivity and phosphorus demand in both site categories. Low phosphorus sites showed strong belowground responses, enhanced microbial phosphorus mobilization, and greater dependence on phosphorus recycling, contributing to progressive phosphorus limitation. In contrast, high‐phosphorus sites maintained a more buffered inorganic phosphorus supply and showed weaker dependence on phosphorus recycling under increasing CO_2_.

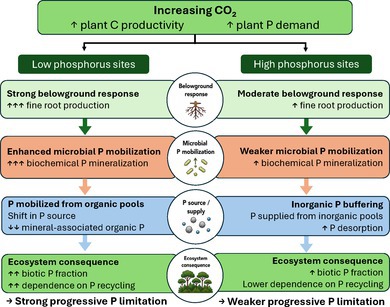

## 1 Introduction

Amazonian tropical forests store substantial amounts of carbon in their aboveground biomass, and their carbon sequestration contributes largely to the contemporary terrestrial carbon sink (Brienen et al. 2015; Friedlingstein et al. 2020; Hubau et al. 2020). Recent observations suggest that the strength of this sink has been declining in recent years (Botía et al. 2025; Brienen et al. 2015; Gatti et al. 2021). Climate change, deforestation, and forest degradation are undermining the forests' capacity to sequester carbon and threaten the long‐term stability of this highly productive ecosystem (Barkhordarian et al. 2019; Feldpausch et al. 2016; Silva Junior et al. 2020). Remarkably, such high productivity is maintained although much of Amazonian forest soils are highly weathered and depleted in rock‐derived nutrients (Darela‐Filho et al. 2024; Quesada et al. 2010). Among these, phosphorus has been experimentally demonstrated to directly constrain primary productivity in mature Amazon forests (Cunha et al. 2022), and to positively influence aboveground wood production across large natural soil nutrient gradients (Quesada et al. 2012). Rising atmospheric CO_2_ concentrations are expected to increase plant phosphorus demand and may therefore intensify nutrient constraints on future forest carbon uptake. Consequently, phosphorus is expected to play a central, yet uncertain, role in regulating the future carbon balance and sequestration potential of Amazonian forests (Fleischer et al. 2019).

In highly‐weathered systems, such as much of tropical soils, rock‐derived nutrients such as phosphorus have become depleted, and are either occluded in inaccessible forms, mineral‐bound, or have been incorporated into organic fractions (Crews et al. 1995; Walker and Syers 1976). Plants have evolved strategies to overcome soil phosphorus scarcity and acquire phosphorus from less available sources, including the exudation of phosphatases and organic acids, and symbioses with mycorrhizal fungi and other microorganisms (Lambers 2022; Wang and Lambers 2020; Wen et al. 2022). Observational studies along natural soil nutrient gradients indicate greater investment in such strategies with increasing nutrient limitation (Reichert et al. 2022; Vicca et al. 2012). Plants may also increase their nutrient‐use efficiency through changes in tissue stoichiometry, altered plant carbon allocation, and greater nutrient retention times (Stuart Chapin et al. 2012). Across Amazonian forests, foliar nutrient concentrations are strongly regulated by soil phosphorus availability, as seen across the RAINFOR plot network (Fyllas et al. 2009). Under iCO_2_, it is expected that increased plant phosphorus demand drives shifts in plant strategies and traits that promote more efficient phosphorus use and enhanced acquisition, with important consequences for forest carbon balance and ecosystem phosphorus cycling (Lugli et al. 2020; Reichert et al. 2022).

Earth System Models (ESMs) generally project that iCO_2_ will enhance tropical forest productivity, partially offsetting the negative impacts of climate change (Koch et al. 2021). However, most ESMs do not incorporate soil phosphorus limitation, which could substantially constrain the CO_2_ fertilization effect on biomass growth. Evidence from small‐scale experiments and process‐based models suggests that phosphorus limitation could reduce tropical biomass gains by up to 50% (Fleischer et al. 2019; Fleischer and Terrer 2022). Phosphorus‐enabled models have incorporated some of the known mechanisms and project that increased belowground allocation and shifts in tissue stoichiometry could sustain CO_2_ fertilization in Amazonian forests despite phosphorus limitation, but the magnitude of this response strongly depended on how flexible these mechanisms were represented (Fleischer et al. 2019; Holm et al. 2020; Yang et al. 2019).

Ecosystems are thought to switch from an acquisition‐based to a recycling‐based nutrient economy through long‐term development, relying increasingly on the mineralization of organic phosphorus (Lambers et al. 2008; Lang et al. 2016). In recycling‐dominated systems, phosphorus availability is governed by the dynamic balance between soil organic pools, plants, microorganisms, and litter (Lang et al. 2016; Peltzer et al. 2010; Turner et al. 2013). In such systems, microbial decomposition of soil organic matter and litter is a key pathway for phosphorus release and plant uptake (Martins et al. 2021; McGroddy et al. 2008; Sayer et al. 2020, 2024; Yu, Ahrens, Wutzler, Zaehle, and Schrumpf 2020). This shift is driven by plant and microbial adaptations, such as reduced leaf nutrient concentrations and longer leaf life spans, which lower nutrient return and alter litter chemistry in nutrient impoverished systems (Peltzer et al. 2010). Consequently, microbial mineralization of phosphorus from litter may decline, reinforcing nutrient limitation—as observed along a tropical chronosequence in Hawaii (Vitousek 1984, 2004). This suggests that during pedogenic development, total phosphorus fluxes among plants, soils, and decomposers may decline, while internal recycling becomes more important as external inputs diminish (Vitousek 2004), though this remains underexplored in other systems (McGroddy et al. 2004, 2008). The degree to which soil phosphorus constrains iCO_2_ responses will thus depend not only on the plants' capacity to meet increased phosphorus demand but also on microbially mediated changes in nutrient cycling, and plant–soil interactions.

Soil enzyme activity reflects the nutrient status of microbial communities. A key indicator of microbial nutrient limitation is the ratio of carbon versus phosphorus acquiring enzymes, or phosphatase release per microbial biomass (Moorhead et al. 2016; Peng et al. 2023). For example, in Central Amazonian forests, the ratio of phosphorus‐ to carbon‐acquiring enzymes varied seasonally with changes in litter input and climate (Schaap et al. 2021). Along the Franz Josef chronosequence in New Zealand, microbial phosphatase activity increased with soil development, suggesting greater microbial phosphorus limitation (Allison et al. 2007). Globally, phosphorus fertilization consistently reduced soil phosphatase activity, as shown by a meta‐analysis (Margalef et al. 2021) and in a long‐term experiment in Panama, where phosphatase activity decreased by 65% and microbial biomass increased after a decade of phosphorus addition (Turner and Joseph Wright 2014). Plant adaptations and soil conditions together shape microbial resource availability, community composition, and nutrient acquisition—while microbial biomass itself represents an important nutrient reservoir in nutrient‐poor systems (Turner et al. 2013).

**FIGURE 1:**
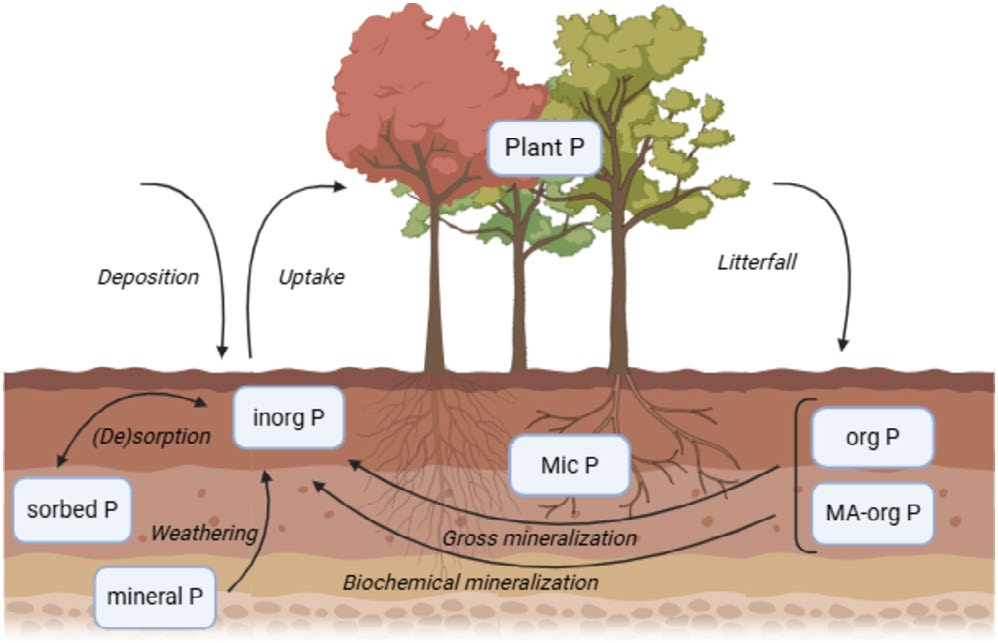
Plant–soil‐microbial phosphorus (P) cycling between plants, soil microorganisms and soil organic and mineral‐associated matter, highlighting modeled mechanisms. Phosphorus is taken up by roots from the plant‐available inorganic phosphorus pool and is incorporated into plant biomass. Phosphorus is returned to the soil via plant litterfall (from roots, leaves and woody material). Soil microbial organisms decompose plant litter and soil organic matter to mineralize phosphorus via the gross mineralization and biochemical mineralization pathway. Mineralized phosphorus is available for microbial uptake (=nutrient immobilization) and for plant uptake from the plant‐available inorganic phosphorus pool. Soil organic phosphorus may be exchanged with mineral surfaces and is partly bound in the mineral‐associated organic matter pool. Phosphorus further enters the plant‐available inorganic phosphorus pool via deposition, (de)sorption dynamic, and weathering. (Figure made with BioRender.com).

In summary, CO_2_ fertilization is expected to increase plant phosphorus demand, potentially intensifying phosphorus limitation. Plants' adjustments in leaf chemistry under iCO_2_ may lead to increases in litter carbon‐to‐phosphorus ratios, altering phosphorus return to soils via litter inputs and reinforcing progressive phosphorus limitation through tighter recycling within the plant–soil–microbe system (Figure 1). In recycling‐dominated tropical soils, phosphorus availability is largely governed by microbial transformation of organic matter and exchanges between organic, inorganic, and mineral‐associated pools (Lang et al. 2016; Turner et al. 2013). Soil microorganisms regulate phosphorus mineralization from organic matter and mediate soil organic matter transformations via necromass production (Liang et al. 2017; Manzoni et al. 2008, 2012; Mooshammer et al. 2012; Sokol and Bradford 2019). Although most soil microbial communities rely on carbon as their primary energy source (Soong et al. 2020), phosphorus availability may constrain microbial growth, increase organic phosphorus turnover (Himeoka et al. 2022), or inhibit microbial oxidation of dissolved organic carbon (Cleveland et al. 2002). Through gross mineralization and biochemical mineralization pathways (Figure 1), soil microorganisms regulate the transfer of phosphorus from organic matter into the plant‐available inorganic pool. Gross mineralization is tightly coupled to carbon turnover, whereas biochemical mineralization via extracellular enzyme production can release phosphorus without concurrent carbon release. Microbial immobilization retains phosphorus in microbial biomass, while inorganic phosphorus exchanges with mineral surfaces via sorption–desorption dynamics. The plant‐available inorganic pool receives inputs from weathering and deposition, comparatively small in highly weathered low‐phosphorus tropical soils (Crews et al. 1995; Walker and Syers 1976) (Figure 1). Together, these microbial‐driven and physicochemical processes govern the fate of carbon and phosphorus entering soils and thereby regulate phosphorus availability and its coupling to soil carbon dynamics, determining how much phosphorus is liberated to sustain plant carbon uptake and how much carbon is stabilized in soils under elevated CO_2_.

A deeper understanding of the processes driving soil phosphorus limitation under elevated CO_2_ in tropical forests, and how plant and microbial traits regulate phosphorus cycling between soil pools in different forms and stabilities is urgently needed. To address this, we simulate plant–soil–microbe biogeochemical dynamics along a soil phosphorus gradient in Amazonian forests using QUINCY‐JSM, a terrestrial biosphere model that explicitly represents carbon, nitrogen, and phosphorus cycling, and includes explicit microbial functions (Thum et al. 2019; Yu, Ahrens, Wutzler, Schrumpf, and Zaehle 2020). QUINCY‐JSM models sink‐driven plant carbon allocation, enabling partitioning between growth and nutrient acquisition, and incorporates microbial‐explicit growth, turnover, and nutrient cycling. It is one of the few terrestrial biosphere models that couple carbon, nitrogen, and phosphorus cycling (Goll et al. 2017; He et al. 2021; Knox et al. 2024; Nakhavali et al. 2022; Yang et al. 2014, 2016; Zhu et al. 2019). The model also includes phosphorus exchange between the soil solution and mineral pools and resolves soil microbial activity along vertical soil profiles (Yu, Ahrens, Wutzler, Schrumpf, and Zaehle 2020; Yu, Ahrens, Wutzler, Zaehle, and Schrumpf 2020). QUINCY‐JSM has previously reproduced observed shifts in ecosystem carbon and phosphorus cycling across temperate beech forests, driven by soil microbial feedbacks (Yu, Ahrens, Wutzler, Zaehle, and Schrumpf 2020).

It is now necessary to test and evaluate phosphorus‐enabled models across tropical soil phosphorus gradients with in situ observations. The forest plots used in this study are wet, old‐growth lowland Amazonian forest and span across major soil types of the Amazon. The sites capture a natural phosphorus gradient that resembles a chronosequence and allows us to apply a space‐for‐time approach to gain insights into long‐term soil development and nutrient cycling (Quesada et al. 2010; Figure S1). We simulate coupled carbon, nitrogen, and phosphorus dynamics between plants, soils, and soil microorganisms under both ambient and increased CO_2_ concentrations from 1901 to 2019. Model initialization is based on field data for soil phosphorus, bulk density, and texture, while observations of plant nutrients and carbon fluxes are used for evaluation. We use these simulations to address the following questions:

1. Carbon and phosphorus cycles along the Amazon soil phosphorus gradient.
  - Does plant carbon belowground allocation increase in low‐phosphorus sites?
  - Do total ecosystem phosphorus fluxes reduce under low‐phosphorus?
  - How do microbial processes contribute to phosphorus release along the gradient?
2. Carbon and phosphorus cycles under increase in CO_2_
  - Does the CO_2_ fertilization effect on tropical biomass depend on soil phosphorus?
  - Can we detect symptoms of progressive phosphorus limitation under iCO_2_?

## 2 Methods

### 2.1 Field Observations Along Natural Soil Phosphorus Gradient

Old‐growth, moist Amazonian forest plots in lowland Amazonia were selected from the RAINFOR forest plot census network (*n* = 31, Table S1). Field observations on forest aboveground biomass growth, loss and standing biomass (Brienen et al. 2015), and soil chemistry and leaf nutrition were available for the selected forest plots (Fyllas et al. 2009; Quesada et al. 2010). The modeled forest plots were selected to be < 500 m in altitude and receiving > 1700 mm of annual precipitation to represent climatic and edaphic conditions in lowland Amazonian moist, old‐growth forests. Based on modeled climate input, the forest plots experience mean annual temperatures of 25.9°C to 28.1°C and cover an annual precipitation gradient of ~2050 and 3330 mm. The selected sites span a measured soil phosphorus gradient from 10 to 730 mg g^−1^ total soil phosphorus, equivalent to 30 to 260 g P m^−2^ in the top 30 cm based, which ranged from 40.6 to 311.8 g P m^−2^ in the simulations (Table S1). In situ measurements of dry matter values were converted to area‐based accounting for in situ soil bulk density; area‐based phosphorus (g P m^−2^) = dry matter‐based phosphorus (mg g^−1^) * bulk density (g cm^−3^) * 10^−3^. The soils span a wide soil texture gradient from 2% to 89% sand, and 1% to 81% clay. We use soil phosphorus Hedley fractions and soil type as indicators for progressing soil development. Fertile soils, such as Gleysols and Fluvisols, have more inorganic plant‐available phosphorus compared to Acrisols and Ferralsols, characterized by low inorganic plant‐available soil phosphorus and higher proportions of potentially residual phosphorus (Figure S1).

### 2.2 Model Description

The process‐based model QUINCY‐JSM is one of the first terrestrial biosphere models that explicitly describes microbial carbon, nitrogen and phosphorus cycling and provides a framework for simulating plant–soil‐microbial interactions in terrestrial ecosystems at half‐hourly timesteps (Thum et al. 2019; Yu, Ahrens, Wutzler, Schrumpf, and Zaehle 2020). The QUINCY model simulates plant growth, photosynthesis and respiration, exchanging carbon with the atmosphere and taking up nitrogen and phosphorus from the soil solution pool. QUINCY simulates dynamic plant allocation to foliage and roots in response to climate and resource availability, and the return of carbon, nitrogen, and phosphorus to the soil via litterfall. The model regulates direct limitations on plant biomass growth through dynamic nonstructural carbohydrate reserves and nutrient recycling (Caldararu et al. 2020; Thum et al. 2019). Plant litter enters litter pools, which are processed by the microbial‐explicit vertically resolved soil model JSM (Yu, Ahrens, Wutzler, Schrumpf, and Zaehle 2020).

JSM considers five distinct soil organic matter pools that each contain carbon, nitrogen, and phosphorus; the microbial biomass, the dissolved organic matter, and the microbial residues pool, of which the last two can be sorbed to mineral surfaces, forming the more available mineral‐associated dissolved organic matter and the more stable mineral‐associated residues pool. JSM considers explicit representations of microbial carbon and nutrient mineralization, as well as uptake and the release through microbial growth and decay, respectively. Microbial decomposition of plant litter and microbial residue releases soil CO_2_ via microbial respiration. Microbial mineralization of organic matter releases nutrients into the soil solution and/or nutrients are directly taken up and immobilized in microbial biomass. The production of soil organic matter occurs via microbial death, which turns microbial biomass into microbial residues. These microbial residues, together with dissolved organic matter, can be adsorbed to mineral surfaces to form mineral‐associated organic matter (Yu, Ahrens, Wutzler, Schrumpf, and Zaehle 2020). The above processes are vertically explicitly resolved in JSM, allowing QUINCY‐JSM to simulate daily and seasonal dynamics in plant and soil carbon, nitrogen, and phosphorus cycling throughout the soil profile.

The phosphorus cycle in QUINCY‐JSM is driven by inorganic and organic fluxes and described in detail elsewhere (Yu, Ahrens, Wutzler, Schrumpf, and Zaehle 2020). Soil phosphorus can enter the plant‐available soil solution pool either by weathering of primary phosphorus, by atmospheric deposition, or by desorption from mineral surfaces. In the soil solution pool, the competitiveness of adsorption and biological uptake of phosphorus by plants and microorganisms is modeled based on the equilibrium chemistry approximation principle (Tang and Riley 2013). Dissolved organic matter (containing carbon, nitrogen, and phosphorus) enters the soil solution via microbial depolymerization of polymeric litter and microbial residues, or via direct inputs of soluble litter. Microbial depolymerization is thus considered the pathway of phosphorus mineralization, which is associated with carbon and nitrogen mineralization. Soil microorganisms take up carbon, nitrogen, and phosphorus from the dissolved organic matter pool and also release carbon, nitrogen, and phosphorus during their decay. Microbial decay leads to direct recycling of carbon, nitrogen, and phosphorus back into the dissolved organic matter pool and to production of organic matter as microbial residues. Soil microbial carbon‐use efficiency (CUE) controls the efficiency in producing microbial biomass from microbial carbon uptake and depends on resource availability. The fraction of carbon returned to the atmosphere via microbial respiration to microbial carbon in biomass production is thus negatively controlled by resource availability. Phosphorus mineralization is also considered to occur ‘biochemically’ and independently of carbon and nitrogen mineralization. This phosphatase enzyme‐based release of phosphorus from organic matter supplies inorganic phosphorus to the soil solution, directly available for plant and microbial uptake. The ‘microbial adaptation approach’ allows for adjustments in microbial enzyme allocation to different pathways for nutrient acquisition depending on nutrient demands and resource availability (Wutzler et al. 2022; Yu, Ahrens, Wutzler, Schrumpf, and Zaehle 2020). Unlike soil microbes, plants can only utilize dissolved inorganic phosphorus in the soil solution through fine root uptake, although plants also contribute to the production of phosphatase and accelerate apatite weathering by an implicit root exudation function (Yu, Ahrens, Wutzler, Zaehle, and Schrumpf 2020).

### 2.3 Model Simulations

The model QUINCY‐JSM is initialized with observed soil characteristics for all sites, including the distribution of soil phosphorus fractions, soil bulk density, and soil texture (Quesada et al. 2010). In this study, we used observed soil phosphorus fractions in the top 30 cm associated with the soil phosphorus pools in JSM as follows: HCl fraction = soil mineral phosphorus pool (apatite); residual fraction = occluded phosphorus; inorganic bicarbonate + resin fraction = labile phosphorus; inorganic hydroxide fraction = slow phosphorus. For all sites, the maximum soil depth was standardized to 9.5 m, with 15 discrete layers, whereby the top 4 layers correspond to the upper 26 cm of soil. Observed top layer soil phosphorus is extrapolated to deeper soil layers for which we lacked observations under the assumption that less‐developed soil types increase soil phosphorus with depth, and highly weathered soils decrease soil phosphorus with depth, for which we considered Gleysols, Fluvisoils, Cambisols to be the less developed soil types and Acrisols, Ferrasols to be highly weathered soils.

Meteorological data from 1901 to 2019 were derived from the CRU JRA data set (version 2.1; Harris 2020), choosing the nearest grid cell for the forest plot locations, extracting daily short and long wave radiation, air temperature, humidity, precipitation, surface pressure, and wind speed and disaggregating to the half‐hourly model time step using a statistical weather generator (Zaehle and Friend 2010). Annual values of atmospheric CO_2_ concentrations (Friedlingstein et al. 2022) as well as nitrogen and phosphorus deposition are further used as model inputs (Thum et al. 2019). Soil parameters for which no in situ measurements were available were derived from SoilGrids for site‐specific locations (Poggio et al. 2021). All sites are simulated as old‐growth broadleaf evergreen tropical forest sites. For this forest type, leaf and fine root N : P vary within prescribed bounds set to 20–35, whereas sapwood, heartwood, and coarse root N : P are assumed invariant. Microbial C : N and N : P are set as constants at 7.6 and 5.6 for all sites (Xu et al. 2013). Two simulation runs from 1901 to 2019 were configured with carbon, nitrogen and phosphorus cycles to test the effect of increasing CO_2_ (280–410 ppm) against one simulation which kept CO_2_ concentrations constant at 280 ppm.

### 2.4 Model‐Data Analysis

We first evaluated model simulations against field observation, such that wood growth dynamics and leaf nutrients QUINCY‐JSM simulations were compared to forest plot observations for the contemporary reference 1999 to 2009. We further analyzed ambient carbon and phosphorus cycling along the soil phosphorus gradient, and the effect of iCO_2_ on carbon and phosphorus cycling was quantified over the historical period between 1901 and 2019 by subtracting the ambient from the high CO_2_ simulation run. Forest plots were categorized into differing soil phosphorus conditions based on top soil phosphorus : low (< 80), mid (> 80 and < 120), and high‐phosphorus (> 120 g P m^−2^) to evaluate the effect of phosphorus in our simulations. The long‐term net carbon change in plant biomass is discussed as plant carbon sequestration in this analysis. We evaluated the model with simulated and observed means, standard deviations, and ranges for all variables for which in situ observations were available. We further computed performance metrics (bias, RMSE, and *R* ^2^) for foliar N : P and aboveground wood production as primary response variables in this study. We statistically compared the effect of iCO_2_ on carbon and phosphorus cycling between the low‐ and high‐phosphorus sites. Group‐level differences were mostly assessed using two‐sample *t*‐tests. We evaluated the normality of each group using Shapiro–Wilk tests. For variables that deviated from normality (Shapiro–Wilk *p* < 0.05), we also performed Wilcoxon rank‐sum tests to check the results.

We further analyzed plant and microbial phosphorus (= biota phosphorus) relative to the actively cycled ecosystem phosphorus, defined as the sum of all phosphorus pools except the passive slow pool, which is unavailable on our modeled timescales. Ecosystem phosphorus turnover was then calculated as the ratio of annual phosphorus mineralization to this actively cycled pool (including plant, litter, microbial, and topsoil phosphorus). The rate of change in biota phosphorus (%) and ecosystem phosphorus turnover (%) over the simulation period was compared among soil phosphorus groups using a linear mixed‐effects model with biota phosphorus and ecosystem phosphorus turnover as the response, centered year and soil phosphorus group as fixed effects, and site as a random intercept to account for variation among sites. The interaction year × soil phosphorus group allowed estimation of soil phosphorus group‐specific slopes, which were extracted with emmeans::emtrends() in R. Tukey‐adjusted pairwise comparisons were used to test for differences in temporal responses among soil phosphorus groups.

## 3 Results

### 3.1 Modeled Plant–Soil‐Microbial Biogeochemistry

**FIGURE 2:**
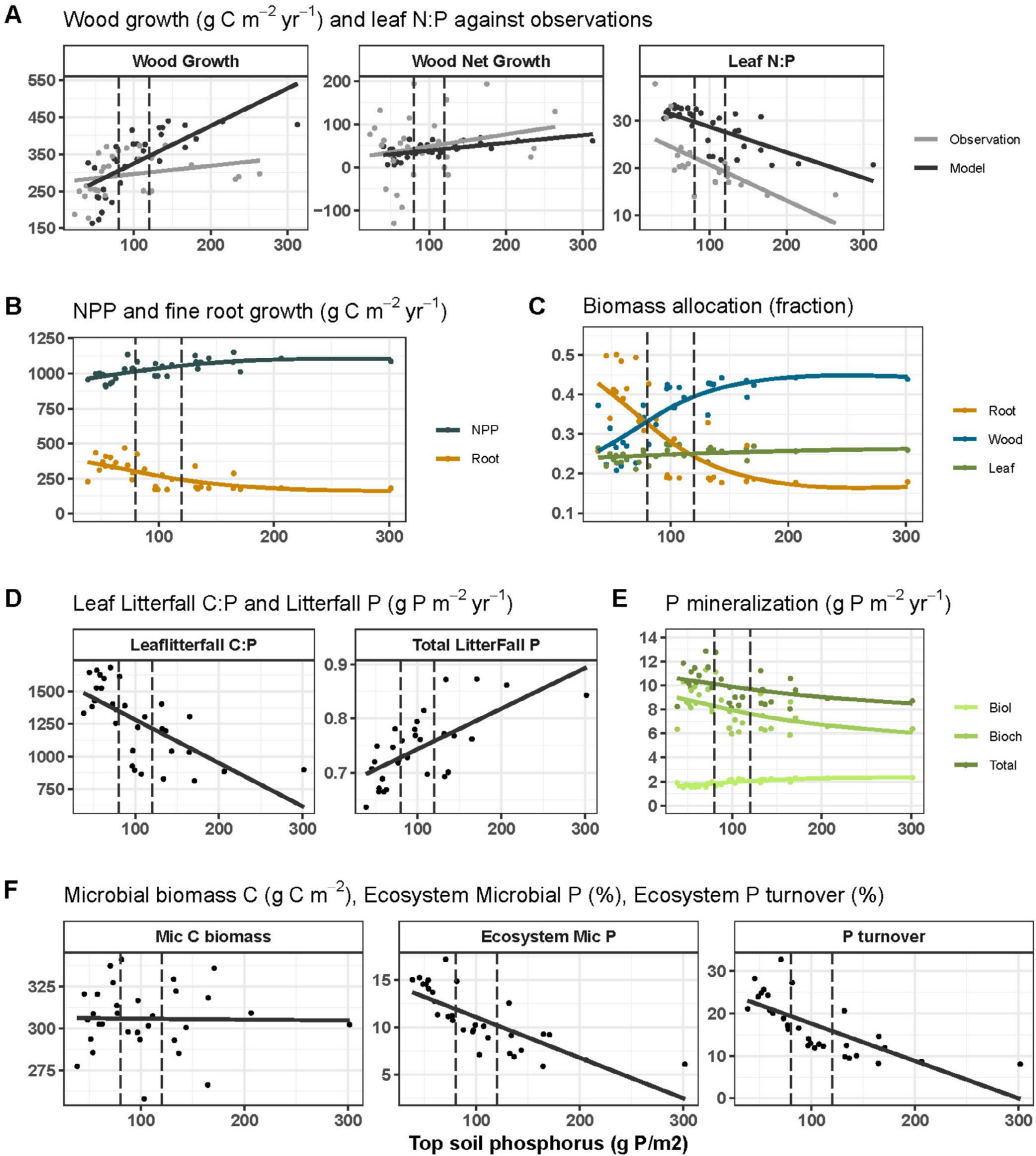
Mean carbon and phosphorus cycling for Amazonian forest plots along the soil phosphorus gradient (g P m^−2^) for the reference period 1999 to 2009, including (A) comparison to forest plot observations on aboveground wood carbon growth, net wood carbon growth (after subtracting wood losses) in g C m^−2^ year^−1^, and leaf nitrogen: Phosphorus ratio, (B) modeled net primary production (NPP) and fine root carbon growth in g C m^−2^ year^−1^, (C) modeled biomass allocation to fine root, wood and leaves (fraction), (D) leaf litterfall carbon: Phosphorus ratio, and total litterfall phosphorus in g P m^−2^ year^−1^, (E) total, gross (=depolymerization) and biochemical phosphorus mineralization in g P m^−2^ year^−1^, and (F) soil microbial biomass carbon in g C m^−2^, microbial phosphorus pools as a percentage of total active ecosystem phosphorus (%), and ecosystem phosphorus turnover as percentage of total active phosphorus pool cycled per year through gross and biochemical phosphorus mineralization fluxes. Modeled values for wood carbon growth combine stem and branch growth, whereas observations reflect stem growth only. Top soil phosphorus reflects total phosphorus in the top four soil layers in model simulations, equivalent to 26 cm soil depth.

The model reproduced the positive relationship between wood growth and soil phosphorus observed in the field but slightly overestimated production and suggested a stronger phosphorus control than observed (Figure 2A). Simulated wood production agreed reasonably well with observations, although values were somewhat higher in the simulations (332 ± 83 vs. 295 ± 54 g C m^−2^ year^−1^; Table 1). Correspondingly, the model showed a positive bias of 36.6 g C m^−2^ year^−1^ and substantial prediction error (RMSE = 90.6), with weak site‐to‐site agreement (*R* ^2^ = 0.10; *n* = 31), reflecting the difficulty of reproducing local variability in stand structure and disturbance history. Net wood carbon gain (growth minus mortality/turnover) was comparable between model and observations during 1999–2019 but more variable in observations (40 ± 18 vs. 47 ± 74 g C m^−2^ year^−1^; Table 1).

**TABLE 1:** Evaluation of QUINCY‐JSM simulated carbon and phosphorus indicators against field observations across Amazonian and pantropical forests, comparing mean, SD, and range (minimum–maximum) for aboveground wood (net) production and stocks, leaf N:P ratios, and fine root allocation, production, and biomass, and soil carbon and soil C : N, with corresponding references.

Variable	Unit	Simulated	Observed	Domain	References
Aboveground wood production	g C m^−2^ year^−1^	332 ± 83 (mean ± SD) 163–440 (range)	295 ± 54 (mean ± SD) 177–374 (range)	Amazon	Brienen et al. (2015)
Aboveground net wood production	g C m^−2^ year^−1^	40 ± 18 (mean ± SD) 6–68 (range)	47 ± 74 (mean ± SD) −130–193 (range)	Amazon	Brienen et al. (2015)
Aboveground wood stock	g C m^−2^	12,900 ± 3020 (mean ± SD) 6500–16,600 (range)	13,000 ± 3050 (mean ± SD) 7400–21,100 (range)	Amazon	Brienen et al. (2015)
Leaf N:P		28 ± 4 (mean ± SD) 20–33 (range)	21 ± 6 (mean ± SD) 14–38 (range)	Amazon	Fyllas et al. (2009)
Fine root allocation	% of NPP	30 ± 11 (mean ± SD) 18–54 (range)	27–31 (range)	Amazon	Aragão et al. (2009); Cordeiro et al. (2020); Malhi et al. (2011)
Fine root production	g C m^−2^ year^−1^ in top soil	273 ± 91 (mean ± SD) 171–461 (range)	300–810 (range) 64–697 (range)	Amazon Pantropical	Aragão et al. (2009); Huaraca Huasco et al. (2021)
Fine root biomass	g C m^−2^ in top soil	187 ± 61 (mean ± SD) 118–317 (range)	200–2400 (range)	Pantropical	Malhi et al. 2009; Huaraca Huasco et al. (2021)
Soil carbon	g C m^−2^ in top soil	6134 ± 1203 (mean ± SD) 3344–8165 (range)	4343 ± 1312 (mean ± SD) 1399–7034 (range)	Amazon	Quesada et al. (2010)
Soil C:N		11 ± 1 (mean ± SD) 8.5–14 (range)	10 ± 2 (mean ± SD) 6.5–16 (range)	Amazon	Quesada et al. (2010)

Leaf N : P ratios decreased along the soil phosphorus gradient in both simulations and observations (Figure 2A), although simulated values were higher and less variable (28 ± 4, range 20–33) than observed values (21 ± 6, range 14–38). Site‐level agreement was moderate (*R* ^2^ = 0.33; *n* = 22), with a positive bias of 6.0 and RMSE of 7.8. Simulated fine‐root allocation, production, and biomass were broadly consistent with published observational ranges (Table 1). Fine‐root allocation represented 30% ± 11% of NPP in the model, within the reported Amazonian range of 27%–31%. Simulated fine‐root production (273 ± 91 g C m^−2^ year^−1^) and biomass (187 ± 61 g C m^−2^) also fell within the wide ranges reported for Amazonian and pantropical forests. The model underestimated standing biomass, particularly at low‐phosphorus sites, and did not reproduce the observed increase in soil C : N ratios at lower fertility (Figure S2). Overall, the model captures the magnitudes and nutrient‐related patterns central to our analysis, although site‐level variability is not fully resolved.

Forest carbon cycling indicators showed signs of phosphorus limitation, expressed as reduced productivity and shifts in carbon allocation and stoichiometry, between soil phosphorus values of 80 and 120 g P m^−2^, which we used to classify forest plots into low‐ (< 80 g P m^−2^, *n* = 13) and high‐phosphorus (> 120 g P m^−2^, *n* = 10) categories. In low‐phosphorus sites, simulated aboveground wood growth was 39% lower compared with high‐phosphorus sites, and net plant carbon gain was 46% lower (74 ± 57 to 39 ± 96 g C m^−2^ year^−1^; Figure 2A, Table 2). Total plant biomass growth, however, was only moderately related to soil phosphorus status. NPP and GPP declined by 9 to 10% across the gradient, while carbon use efficiency remained stable (Table 2). However, phosphorus limitation strongly shifted carbon allocation patterns: fine root growth was 68% higher (Figure 2B), and fine root biomass was 65% greater in low‐phosphorus sites (Table 2). The higher investment in fine root growth occurred mainly at the expense of stem and leaf growth, such that the relative allocation (fraction of NPP) shifted from fine roots (low‐phosphorus) to wood (high‐phosphorus) along the gradient (Figure 2C). In the model, flexible tissue stoichiometry and allocation allowed plants to maintain productivity despite phosphorus constraints.

**TABLE 2:** Modeled plant carbon and phosphorus cycling in QUINCY‐JSM simulations for Amazonian forest sites.

	High P sites mean	SD	Low P sites mean	SD	P limitation (%)
GPP	3729.23	125.76	3351.18	331.07	−10.14
NPP	1086.88	65.11	987.03	116.35	−9.19
Plant CUE	0.29	0.02	0.29	0.03	0
Plant C stock	17640.91	1347.37	12086.47	2565.69	−31.49
Plant C net change	73.56	56.78	39.16	95.73	−46.76
C fine root stock	143.73	37.42	237.72	45.57	65.39
C fine root growth	208.5	54.18	349.58	69.6	67.66
C leaf growth	256.43	9.29	214.69	21.05	−16.28
C sapwood growth	238.44	43.18	145.36	78.1	−39.04
C heartwood growth	185.81	16.9	119.96	28.85	−35.44
C litterflux to soil	1002.37	30.68	935.74	56.67	−6.65
Plant C residence time (years)	18.87	1.36	13.83	2.42	−26.71
Plant P uptake	0.82	0.11	0.72	0.1	−12.2
Leaf N:P	25.35	4.25	31.75	1.07	25.25
Plant PUE	1356.48	192.8	1382.82	129.24	1.94
Microbial C stock	306.3	27.25	308.1	19.8	0.59
Microbial CUE (top)	0.59	0.01	0.57	0.02	−3.39
Microbial P (top + deep)	7.19	0.64	7.23	0.46	0.56
Soil organic C (top)	6703.71	680.27	5786.59	1298.14	−13.68
Soil organic C (deep)	25375.13	4147.59	21792.38	7300.52	−14.12
Soil organic C:P (top)	101.61	17.57	164.93	31.9	62.32
Plant P stock	5.23	0.5	3.43	0.7	−34.42
P litterflux to soil	0.79	0.07	0.7	0.05	−11.39
Litterflux C:P	1278.52	133.09	1333.09	63.97	4.27
Leaf Litterflux C:P	1063.49	207.15	1496.47	147.75	40.71
Plant P residence time (years)	7.64	0.78	5.7	1.1	−25.39
P depoly. mineralization	2.16	0.17	1.71	0.16	−20.83
P biochem. mineralization	7.06	1.19	8.94	1.27	26.63
P total mineralization	9.23	1.15	10.64	1.19	15.28
P weathering	0.6	0.45	0.08	0.09	−86.67
P fast exchange	−0.41	0.41	−0.06	0.13	−85.37
P slow exchange	−0.17	0.49	−0.02	0.07	−88.24

*Note:* Mean and standard deviation of pools, fluxes, and other stoichiometric variables aggregated for 2000 to 2019, separated by low and high soil phosphorus sites. The effect of phosphorus limitation is shown as the difference between low and high phosphorus sites in %. Carbon and phosphorus pools and fluxes are in g C/P m^−2^ (year^−1^). Soil carbon pools are separated for top soil layers (0–26 cm) and deep soil layers (26 cm–9.5 m). Fast and slow exchange of phosphorus are net sorption fluxes to mineral surfaces when negative.

Despite lower plant growth, litter production was only 7% lower in low‐phosphorus sites, sustained by higher fine root litter inputs causing faster turnover of plant carbon (Table 2, Figure S3). Carbon was increasingly routed belowground through short‐lived roots under phosphorus limitation. Leaf litter C : P ratios were 40% lower in low‐phosphorus sites, while litter phosphorus flux was 11% lower (Table 2, Figure 2D), indicating disproportionate reductions in phosphorus cycling between plants and soils compared to carbon.

Phosphorus mineralization in low‐phosphorus sites was mainly sustained through the biochemical mineralization pathway (i.e., through phosphatases). Total phosphorus mineralization rates were 15% higher in low‐phosphorus sites, driven by 27% higher biochemical mineralization rates (8.9 ± 1.3 vs. 7.1 ± 1.2 g P m^−2^ year^−1^; Figure 2E, Table 2). In contrast, mineralization via depolymerization was 21% lower in low‐phosphorus sites due to lower litter phosphorus content, which limited phosphorus release and drove greater microbial enzyme investment into biochemical processes. Higher biochemical mineralization was simultaneously driven by higher fine root biomass in low‐phosphorus sites.

Soil microbial biomass remained relatively stable across the phosphorus gradient (Figure 2F, Table 2). Microbial CUE declined slightly from 0.59 ± 0.01 to 0.57 ± 0.02 due to increased respiration costs from greater investment in biochemical mineralization. Soil microorganisms maintained similar levels of phosphorus and nitrogen uptake through increased reliance on inorganic phosphorus released via biochemical mineralization. Microbial biomass accounted for a larger share of ecosystem phosphorus in low‐phosphorus sites, rising from just above 5% to > 15% (Figure 2F). As phosphorus availability declined, total mineralization increased, leading to greater ecosystem phosphorus turnover (rising from ~10 to > 30%) to fulfill biological demand in low‐phosphorus systems (Figure 2F).

**FIGURE 3:**
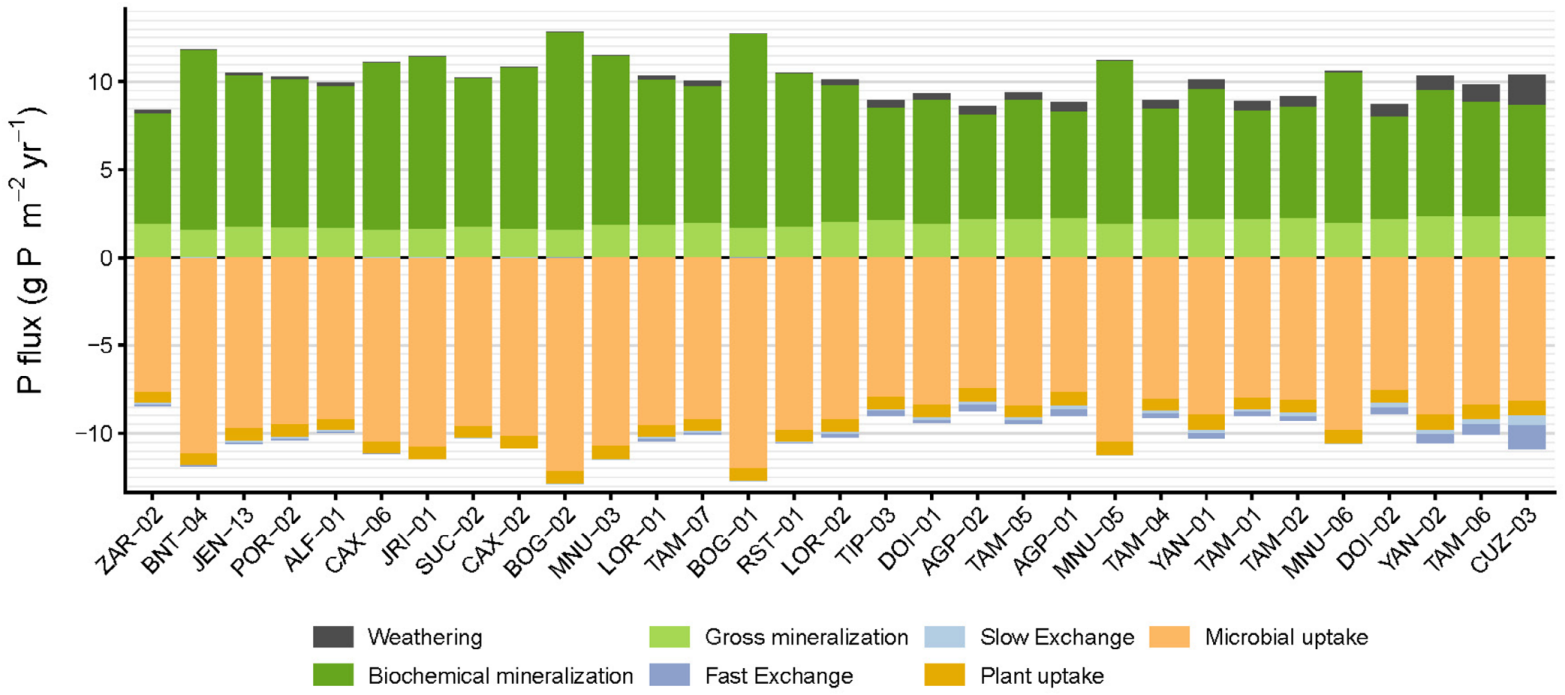
Modeled soil solution phosphorus budget in g P m^−2^ year^−1^ for Amazonian forest plots along the soil phosphorus gradient, sorted by top soil phosphorus availability from left to right. Influxes to the plant‐available soil solution phosphorus pool are positive, including weathering, biochemical mineralization, and gross mineralization (=depolymerization). Outfluxes from the plant‐available soil phosphorus pool are negative, including plant and microbial phosphorus uptake. Fast and slow exchange of phosphorus are net sorption fluxes to mineral surfaces when negative. Phosphorus fluxes are in g P m^−2^ year^−1^ and aggregated for the entire 9.5 m soil column for the years 2000 to 2019.

Phosphorus mineralization is the dominant input pathway to the soil solution phosphorus and contributed more than 80% in all forest sites, and there was a growing reliance on internal recycling of organic phosphorus in low‐phosphorus systems (Figure 3). Inorganic inputs via weathering were negligible in low‐phosphorus sites (0.08 ± 0.09 g P m^−2^ year^−1^) and relatively low in high‐phosphorus sites (0.6 ± 0.45 g P m^−2^ year^−1^; Table 2). Sorption/desorption fluxes were in equilibrium and contributed minor net outputs, equivalent to weathering inputs in both low‐ and high‐phosphorus sites (Figure 3, Table 2). Total phosphorus inputs were in fact higher in low‐phosphorus sites than in high‐phosphorus sites, and phosphorus mineralization contributed on average 88.5% in high‐phosphorus sites, and 98.5% in the low‐phosphorus sites (Figure 3, Table 2).

### 3.2 Modeled CO_2_ Fertilization Effect

**FIGURE 4:**
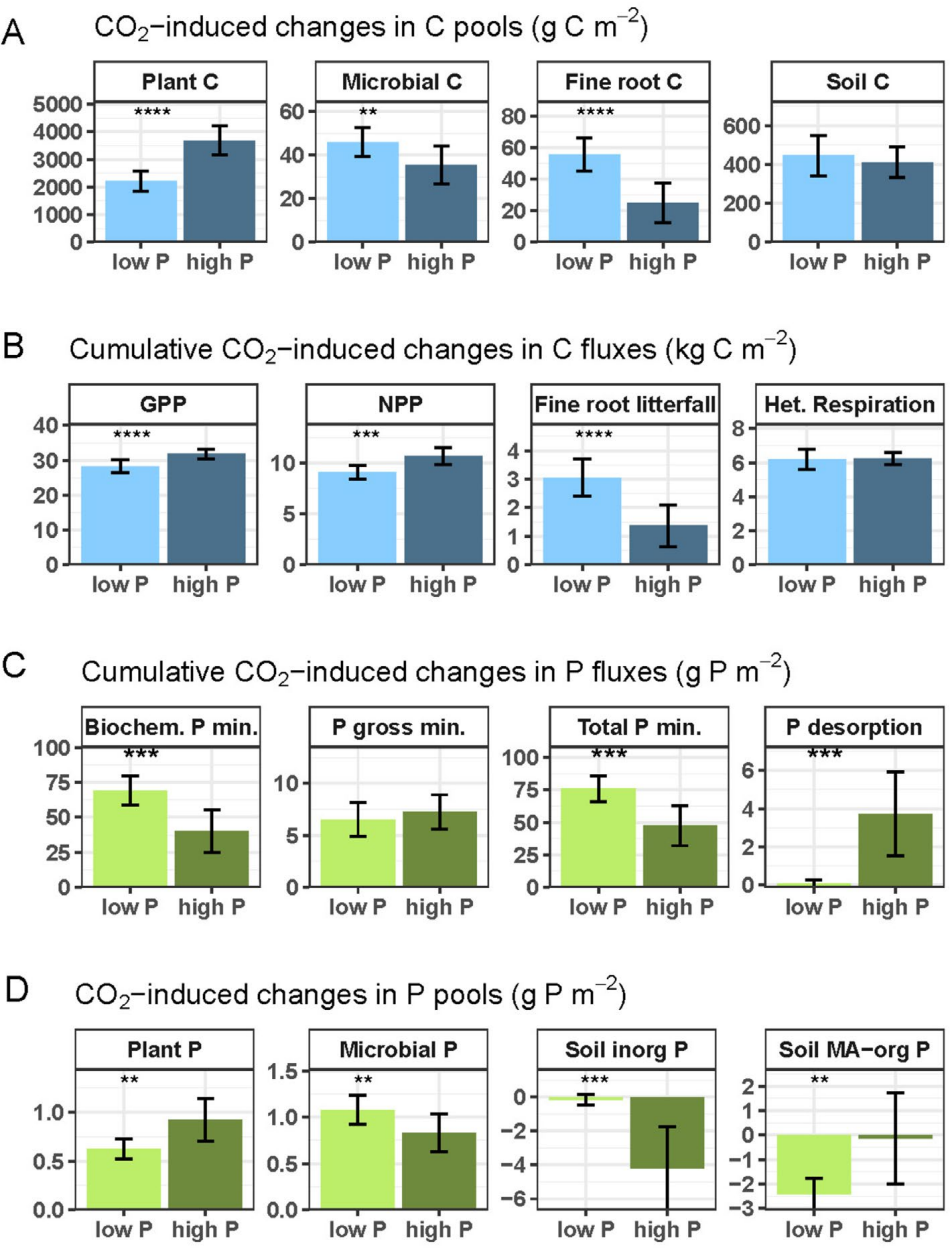
Modeled iCO_2_ effects on plant, litter, soil, and soil microorganisms' carbon and phosphorus stocks and cumulative fluxes in (k)g C/P m^−2^ for Amazonian forest plots. Pools and fluxes are summarized over the last 3 years of the simulation, and cumulative fluxes are calculated after the entire simulation period. CO_2_ effects are shown as mean and SD for forest sites grouped by low (*n* = 13) and high top soil phosphorus content (*n* = 10), including (A) change in carbon stock in plants, litter, microbial biomass, fine roots, and soil, (B) cumulative fluxes for GPP, NPP, fine root litter flux, and heterotrophic respiration, (C) cumulative fluxes of biochemical, gross (biological), and total phosphorus mineralization and phosphorus desorption, and (D) change in phosphorus stock in plants, microbial biomass, soil inorganic and soil mineral‐associated organic pool. Group differences were assessed using two‐sample *t*‐tests. Significance of this test is indicated with asterisks (*p < 0.05, **p < 0.01, ***p < 0.001). Positive values for phosphorus desorption indicate a flux of phosphorus from the sorbed inorganic pool to the soil solution pool.

The CO_2_ fertilization effect induced a 23.3% increase in plant carbon across all forest sites, reaching an average of 2879 ± 771 g C m^−2^ by the end of the simulation (Table 3). However, low‐phosphorus sites accumulated 40% less additional carbon than high‐phosphorus sites (Figure 4A, Table 3), which was mainly driven by lower initial plant carbon in low‐phosphorus sites, while the relative CO_2_ effects were comparable (25% vs. 22% under low‐phosphorus; Table 3). The iCO_2_ effect on gross and net primary production was similar across high‐ and low‐phosphorus sites, with cumulative increases of 32 ± 1 and 28 ± 2 kg C m^−2^, respectively (Figure 4B, Table 3). Despite low‐phosphorus availability, flexible tissue stoichiometry enabled increased production under iCO_2_ in low‐phosphorus sites. Carbon allocation shifted toward roots, increasing fine‐root biomass by 29.5% under iCO_2_ and causing cumulative fine‐root litter inputs to more than double relative to high‐phosphorus sites (Figure 4B, Table 3). Differences in CO_2_‐induced changes in carbon pools and cumulative carbon fluxes between high‐ and low‐phosphorus sites were significant for all variables reported above (two‐sample *t*‐tests, *p* < 0.05; Table S2).

**TABLE 3:** Modeled iCO_2_ effects on plant, litter, soil, and soil microorganisms' carbon and phosphorus stocks and fluxes, and cumulative sums of fluxes in (k)g C/P m^−2^ for Amazonian forest plots.

	All sites mean	All sites SD	All sites mean %	Low sites mean	Low sites SD	Low sites mean %	High sites mean	High sites SD	High sites mean %
Plant C	2879.1	770.9	23.3	2208.1	371.9	22.4	3690.0	537.7	25.4
Litter C	371.1	124.6	8.7	328.7	76.7	8.3	470.9	119.1	10.2
Microbial C	39.7	10.1	14.8	46.0	6.7	17.2	35.4	8.6	12.9
Fine root C	39.6	19.6	24.4	55.4	10.6	29.5	25.0	12.6	19.9
Soil C	411.4	123.8	1.5	445.3	104.4	1.7	410.6	79.5	1.3
Plant P	0.8	0.2	22.0	0.6	0.1	22.4	0.9	0.2	20.9
Litter P	0.2	0.1	8.3	0.1	0.0	8.8	0.2	0.1	8.5
Microbial P	0.9	0.2	14.8	1.1	0.2	17.2	0.8	0.2	12.9
Soil inorg P	−1.9	2.3	0.0	−0.2	0.3	0.1	−4.2	2.5	0.0
Soil MA‐org P	−1.3	1.8	−0.7	−2.4	0.6	−1.4	−0.1	1.9	−0.1
Biochem. P min.	1.4	0.6	20.6	1.8	0.3	24.5	1.1	0.4	17.2
Depoly. P min.	0.2	0.1	10.2	0.2	0.1	11.2	0.2	0.0	9.4
P desorption	0.0	0.1	85.1	0.0	0.0	183.6	0.1	0.1	16.9
P weathering	0.0	0.0	1.6	0.0	0.0	2.5	0.0	0.0	0.7
GPP (sum)	30.1	2.5	NA	28.2	1.8	NA	31.9	1.4	NA
NPP (sum)	9.7	1.0	NA	9.1	0.7	NA	10.6	0.8	NA
Fine root litterfall (sum)	2.2	1.1	NA	3.1	0.7	NA	1.4	0.7	NA
Het. respiration (sum)	6.1	0.5	NA	6.2	0.6	NA	6.3	0.3	NA
Biochem. P min. (sum)	54.1	19.2	NA	69.3	10.1	NA	40.1	15.2	NA
P gross min. (sum)	6.9	1.7	NA	6.5	1.7	NA	7.3	1.7	NA
Total P min. (sum)	61.0	18.8	NA	75.8	9.7	NA	47.4	15.3	NA
P desorption (sum)	1.6	2.2	NA	0.1	0.2	NA	3.7	2.2	NA
P weathering (sum)	0.0	0.1	NA	0.0	0.0	NA	−0.1	0.1	NA

*Note:* Pools and fluxes are summarized over the last 3 years of the simulation, and cumulative fluxes are calculated after the entire simulation period. CO_2_ effects are shown as mean and SD for all forest sites, and forest sites grouped by low and high soil phosphorus availability. Microbial and soil pools represent the entire soil column, thus the sum of top and deep layers. CO_2_‐induced changes in carbon and phosphorus pools and fluxes are additionally shown as relative change to initial conditions.

In contrast, soil carbon sequestration and cumulative heterotrophic respiration did not differ between phosphorus conditions. Litter, soil microorganisms, and soil organic matter pools all gained carbon under iCO_2_, although changes were modest and were largely independent of soil phosphorus (Figure 4A, Table 3). Soil carbon sequestration was similar between low‐ and high‐phosphorus sites (Figure 4B, Table 3), as greater litter production per unit growth in low‐phosphorus sites compensated for moderately lower primary productivity (Figure S4). Cumulative heterotrophic soil respiration under iCO_2_ was likewise similar between low‐ and high‐phosphorus sites, averaging 6.1 ± 0.5 kg C m^−2^ across all sites (Figure 4B, Table 3).

Enhanced litter inputs under elevated CO_2_ stimulated both depolymerization and biochemical phosphorus mineralization across all forest sites, increasing annual rates at the end of the simulation by 10% and 21% on average, respectively (Table 3). Over the entire simulation, phosphatase‐driven mineralization supplied the majority of phosphorus release under iCO_2_, accumulating to 69 ± 10 g P m^−2^ in low‐phosphorus and 40 ± 15 g P m^−2^ in high‐phosphorus sites (Figure 4C, Table 3). Low‐phosphorus sites therefore saw a 58% higher enhancement of phosphatase‐driven mineralization. In contrast, cumulative gross mineralization of inorganic phosphorus increased much less and similarly across the phosphorus gradient, averaging 7 ± 2 g P m^−2^ across all sites (Figure 4C, Table 3). Lower litter quality under phosphorus limitation intensified microbial reliance on the biochemical mineralization pathway to access organic phosphorus. Inorganic phosphorus fluxes were also much less enhanced under iCO_2_, with cumulative desorption reaching 3.7 ± 2.2 g P m^−2^ in high‐phosphorus sites and near zero in low‐phosphorus sites (0.01 ± 0.16 g P m^−2^; Figure 4C, Table 3). Similarly, iCO_2_‐induced phosphorus input from weathering was negligible across all sites. Overall, 95% of the additional phosphorus under iCO_2_ was supplied through organic mineralization, while only 5% came from desorption and weathering (Table 3). All reported differences in CO_2_‐induced changes of cumulative phosphorus fluxes between high‐ and low‐phosphorus sites were significant, except gross mineralization (two‐sample *t*‐tests, *p* < 0.05; Table S2).

Plants benefited from increased phosphorus mineralization under iCO_2_, with phosphorus uptake rising across all sites and most strongly in low‐phosphorus sites due to the initially lower availability (Table 3). Cumulative phosphorus uptake under iCO_2_ was higher in low‐phosphorus sites since the growth of root tissues with faster turnover increased phosphorus demand (Figure S5). For that reason, plant phosphorus residence times under iCO_2_ decreased in low‐ but not in high‐phosphorus sites (Figure S6). The increased investment in roots under iCO_2_ and nutrient limitation thus led to faster plant phosphorus turnover.

Increased CO_2_ shifted phosphorus from soil pools to living organisms, driven by increased biological phosphorus demand from both plants and soil microorganisms. Across all sites, plant phosphorus increased by 0.8 ± 0.2 g P m^−2^ and microbial phosphorus by 0.9 ± 0.2 g P m^−2^ (Table 3, Figure 4D). The source of that phosphorus differed between high‐ and low‐phosphorus sites. In high‐phosphorus sites, most demand was met by depleting inorganic soil phosphorus (−4.2 ± 2.5 g P m^−2^), whereas in low‐phosphorus sites, plants relied on mobilization from organic pools, especially from the mineral‐associated residue pool (−2.4 ± 0.6 g P m^−2^; Table 3, Figure 4D). Consequently, nearly all phosphorus gains in plant and microbial biomass in low‐phosphorus sites were supplied via microbial‐driven mineralization. All reported differences in CO_2_‐induced changes of phosphorus pools between high‐ and low‐phosphorus sites were significant (two‐sample *t*‐tests, *p* < 0.05; Table S2).

**FIGURE 5:**
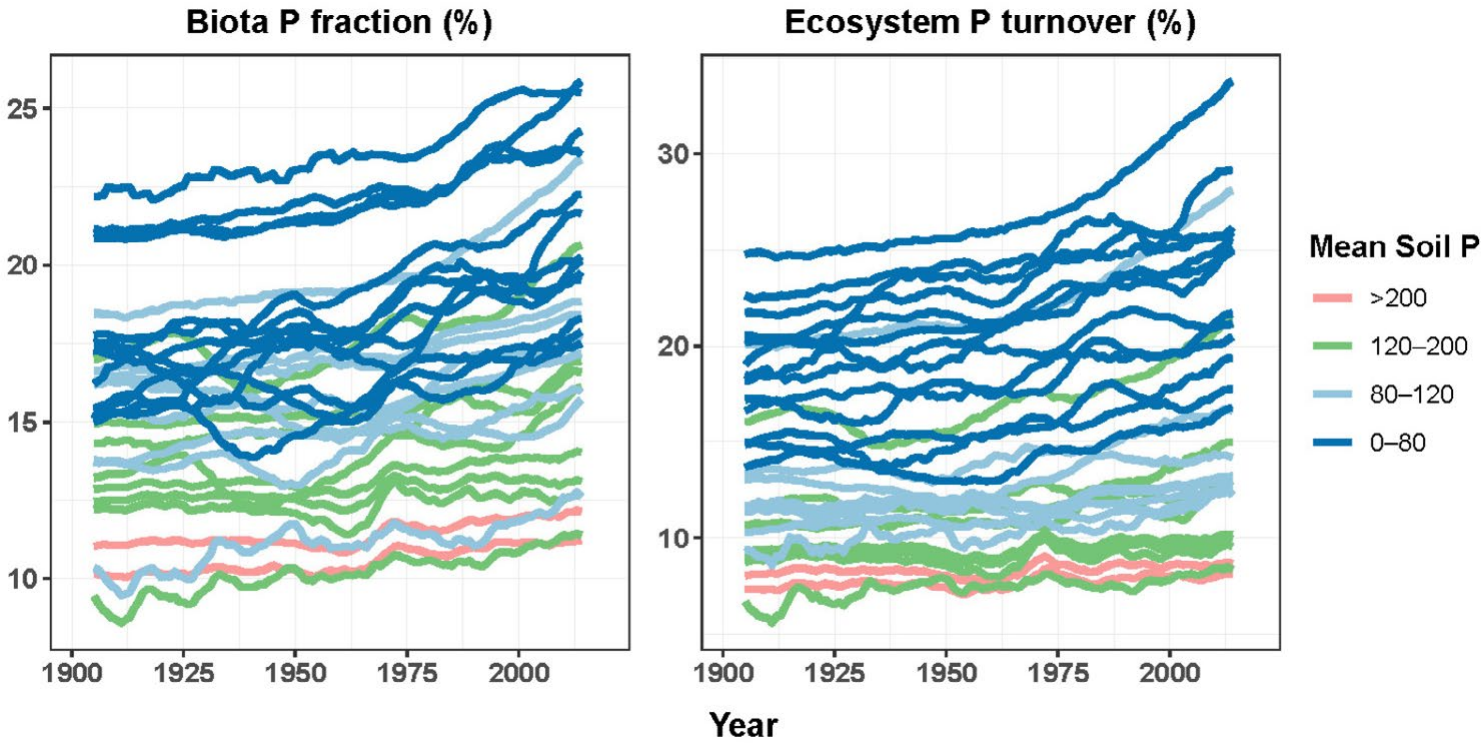
Temporal dynamics of biotic phosphorus fraction (%) and ecosystem phosphorus turnover (%) across soil phosphorus categories. Ten‐year centered rolling means of (left) biotic phosphorus fraction and (right) ecosystem phosphorus turnover are shown for all sites. Biota phosphorus fraction represents the sum of microbial and plant phosphorus pools expressed as a percentage of total active phosphorus in the ecosystem (excluding non‐active pools). Ecosystem phosphorus turnover represents the percentage of the active phosphorus pool cycled per year through gross and biochemical phosphorus mineralization fluxes. Lines represent individual sites and are color‐coded according to soil phosphorus group; low = 0–80 (*n* = 13), mid = 80–120 (*n* = 8), high = 120–200 (*n* = 8), very high = > 200 (*n* = 2), defined based on the topsoil phosphorus at the end of the simulation period (2000–2019). Rolling means were calculated to reduce interannual variability.

**FIGURE 6:**
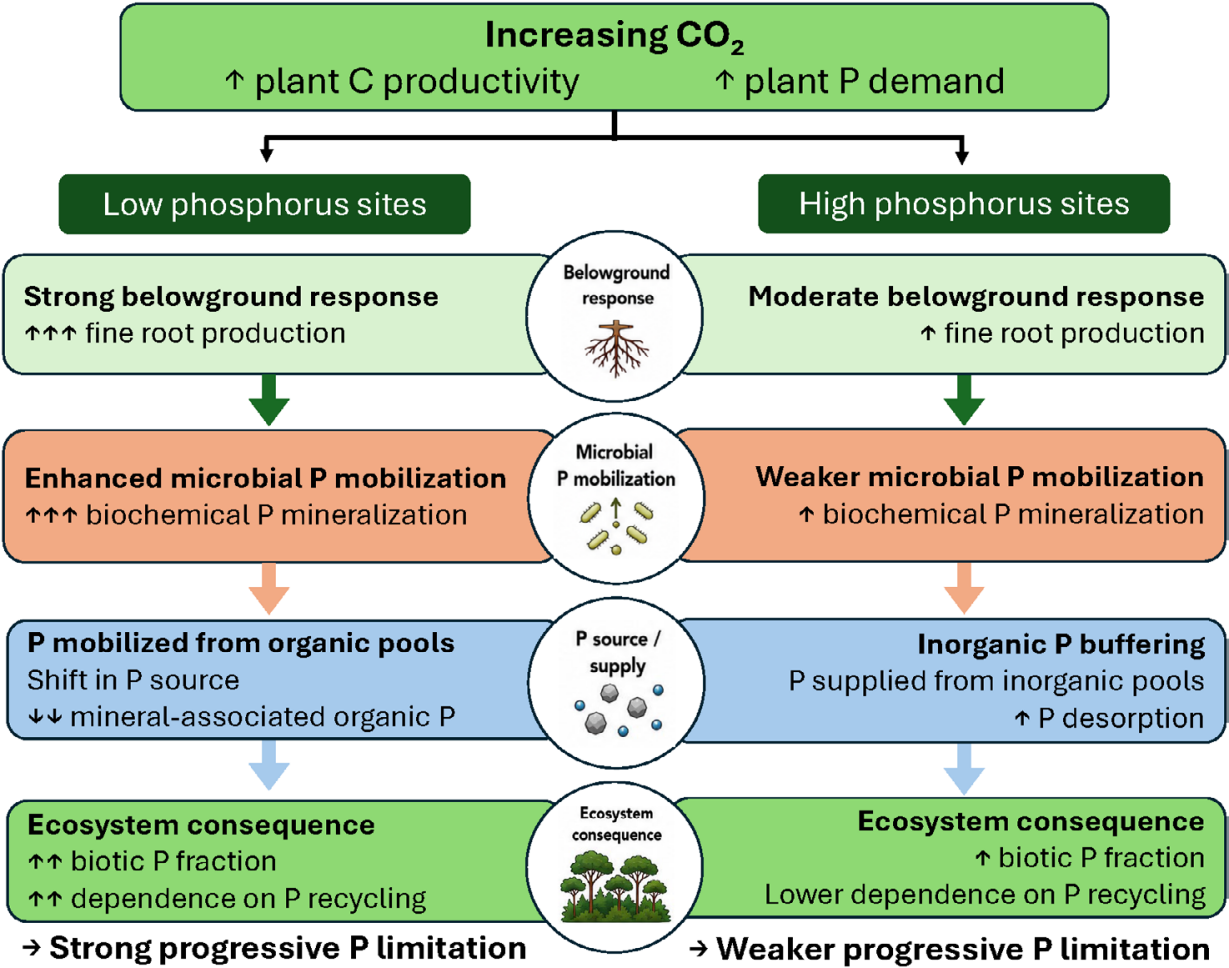
Conceptual synthesis of plant response and phosphorus redistribution pathways under increasing CO_2_ in low and high phosphorus Amazonian forest sites. Increasing CO_2_ led to higher plant productivity and phosphorus demand in both site categories. Low phosphorus sites showed strong belowground responses, enhanced microbial phosphorus mobilization, and greater dependence on phosphorus recycling, contributing to progressive phosphorus limitation. In contrast, high‐phosphorus sites maintained a more buffered inorganic phosphorus supply and showed weaker dependence on phosphorus recycling under increasing CO_2_.

This shift increased the role of living organisms as a phosphorus reservoir, progressively raising the fraction of ecosystem phosphorus stored in plants and microorganisms over time (Figure 5). In parallel, ecosystem phosphorus turnover increased, indicating a growing reliance on phosphorus recycling. The rate of change in both variables was positive regardless of soil phosphorus conditions, but significantly higher in the low‐phosphorus groups. For example, the biotic phosphorus fraction increased from 17.9% to 21.3% in the low‐phosphorus group compared to 13.3% to 15.2% in the high‐phosphorus group (Table 4). Similarly, ecosystem phosphorus turnover increased from 18.7% to 23.7% in low‐phosphorus sites but only from 10.4% to 12.5% in high‐phosphorus sites. Slopes increased systematically from high‐ to low‐phosphorus groups (Table 4), and pairwise comparisons (Tukey‐adjusted) confirmed that slopes differed significantly among all soil phosphorus groups (Table S3), providing a clear signal of progressive phosphorus limitation under iCO_2_. The contrasting plant response and phosphorus redistribution pathways under increasing CO_2_ are summarized conceptually in Figure 6.

**TABLE 4:** Changes in biotic phosphorus fraction (%) and ecosystem phosphorus turnover (%) across soil phosphorus groups over the simulation period (1901–2020).

Soil P group	Start biota P (%)	End biota P (%)	Slope (%/year)	120‐year change	Slope group
0–80	17.9	21.3	0.03	3.57	a
80–120	15.1	17.5	0.02	2.36	b
120–200	13.3	15.2	0.015	1.81	c
> 200	10.6	11.7	0.011	1.25	d
	**Start P turnover (%)**	**End P turnover (%)**			
0–80	18.7	23.7	0.044	5.19	a
80–120	12.6	15.3	0.023	2.69	b
120–200	10.4	12.5	0.016	1.88	c
> 200	7.7	8.4	0.006	0.66	d

*Note:* Values show the mean during the first (“Start”) and last (“End”) 10 simulation years, the estimated temporal slope from linear mixed‐effects models, and the total change over the 120‐year simulation. Slopes represent annual rates of change. Biotic phosphorus fraction indicates the percentage of ecosystem phosphorus contained in plant and microbial biomass, while ecosystem phosphorus turnover represents the annual percentage of the active phosphorus pool cycled through mineralization fluxes.

Phosphorus in the mineral‐associated residue pool was reduced in the top layers due to greater phosphorus demand by plants and soil microorganisms, indicating growing soil phosphorus scarcity under iCO_2_ across the entire gradient (Figure S7). In high‐phosphorus sites, iCO_2_ induced an increase in soil organic phosphorus due to greater productivity, mainly in the residues pool in both top and deep layers and the mineral‐associated residues pool in the deep layers (Figure S7).

## 4 Discussion

Our simulations show that phosphorus limitation alters plant carbon allocation in tropical forests, with low‐phosphorus conditions shifting carbon toward fine roots. Flexible tissue stoichiometry and microbial‐mediated mineralization allow forests to maintain productivity despite nutrient scarcity, supporting a tight phosphorus cycle. Contrary to the expectation of reduced phosphorus cycling, our model indicates an increase in ecosystem phosphorus turnover in low‐phosphorus sites, including the depletion of organic phosphorus pools, particularly less‐accessible mineral‐associated residues. Elevated CO_2_ increased plant carbon, but gains are smaller in low‐phosphorus sites, reflecting constraints on wood production and prioritized allocation to fast‐turnover tissues.

Soil microorganisms play a central role in sustaining phosphorus availability by accelerating soil organic phosphorus mineralization. As CO_2_ enrichment continues, progressive phosphorus limitation may increasingly constrain forest carbon gains, highlighting that tropical forest carbon sequestration depends on tightly coupled plant, microbial, and soil organic phosphorus dynamics.

### 4.1 Soil Phosphorus Limitation on Plant Carbon Dynamics

The model simulated reduced aboveground wood production under low‐phosphorus conditions, consistent with field observations across Amazonian forests (Muller‐Landau et al. 2021; Quesada et al. 2012) and rarely reproduced by previous phosphorus‐enabled vegetation models (but see e.g., Yang et al. 2019). The model applied a resource‐dependent allocation scheme (Bloom et al. 1985; Thum et al. 2019), which postulated that phosphorus limitation shifts carbon allocation from stems toward fine roots. In our simulations, relative fine root allocation was 19% in high‐phosphorus sites and 35% in low‐phosphorus sites. Fine root production and biomass were substantially higher in low‐phosphorus sites, exceeding those in high‐phosphorus sites by 68% and 65%, respectively. Relative allocation, fine root production and biomass all fall within observational ranges across Amazonian and pantropical forests (Table 1) (Aragão et al. 2009; Cordeiro et al. 2020; Huaraca Huasco et al. 2021; Malhi et al. 2009; Malhi et al. 2011), though often at the lower end suggesting even greater fine root production and biomass are reasonable.

Notably, some field studies reported the opposite pattern, with higher fine root production in high‐phosphorus soils (Aragão et al. 2009), and fertilization experiments in the Central Amazon confirmed that phosphorus limits both canopy and root growth (Cunha et al. 2022). Root productivity is also influenced by soil texture, water availability, and chemical constraints in highly‐weathered soils, such as low pH or aluminum toxicity (Huaraca Huasco et al. 2021), which our model does not capture, nor does it include carbon investments in alternative acquisition strategies like mycorrhizae or root exudates (Reay et al. 2025; Reichert et al. 2022; Wen et al. 2022). While increased belowground allocation under low‐phosphorus conditions is plausible, the magnitude of simulated phosphorus limitation and associated reductions in aboveground productivity may depend on the efficiency of alternative phosphorus acquisition pathways. However, most models cannot yet represent the relative carbon costs and trade‐offs between root growth and other acquisition strategies or the multiple constraints on root expansion, though some recent efforts begin to explore these dynamics (Braghiere et al. 2022; de Paula et al. 2025; Schufft et al. 2026).

The model further predicted that plant carbon residence times decreased under phosphorus limitation, whereas field data more commonly suggested longer tissue lifespans under nutrient stress. Stable aboveground turnover rates in the model prevented reproduction of observed increases in turnover along soil physical and chemical gradients (Castanho et al. 2013; Quesada et al. 2012; Yang et al. 2019), so aboveground biomass did not reach the very high values observed in some low‐phosphorus sites in the Amazon. Despite these simplifications, the model captured the primary mechanism by which belowground investment buffers productivity under phosphorus scarcity. CO_2_ fertilization effects were dampened in low‐phosphorus sites due to constraints on wood production and allocation to fast‐turnover tissues, illustrating how nutrient limitation moderated forest carbon gains.

### 4.2 Ecosystem Phosphorus Cycling

Our model results suggested a downregulation of the plant phosphorus cycle as an adaptive response to nutrient scarcity. Litter phosphorus return declined under low‐phosphorus conditions, driven by lower productivity and higher leaf N : P ratios. The simulated changes in tissue stoichiometry are consistent with observations across Amazonian forest plots (Fyllas et al. 2009), though reductions in plant–soil phosphorus cycling may be underestimated, as longer tissue lifespans and higher phosphorus resorption under low‐phosphorus conditions would further reduce litter phosphorus return (Cunha et al. 2022; Reichert et al. 2022).

While plant–soil cycling declined under low‐phosphorus, soil phosphorus cycling increased, with a clear shift in supply pathways. Organic phosphorus mineralization dominated soil solution inputs across the gradient, whereas high‐phosphorus sites also relied on geochemical inputs such as weathering and desorption (Cleveland et al. 2013; Yang and Post 2011). At low‐phosphorus sites, geochemical inputs were minimal, and phosphorus availability depended increasingly on biochemical mineralization, which was 24% higher under low‐phosphorus due to greater plant and microbial demand. This supports the idea that microbial and plant communities adaptively enhance phosphatase‐driven mineralization to maintain function under strong nutrient limitation (Martins et al. 2021; Pistocchi et al. 2018; Schaap et al. 2021).

These dynamics align with empirical evidence of increased phosphatase activity under low‐phosphorus (Margalef et al. 2017; Marklein and Houlton 2012), and seasonal coupling has been observed between phosphatase activity and soil organic phosphorus fractions in Central Amazon soils (Schaap et al. 2021). However, interpreting phosphatase activity as a direct proxy for mineralization remains complex, since organic pools vary in accessibility, and phosphatase activity may also reflect carbon demands (McConnell et al. 2020). Nevertheless, the model reproduces the trend toward a more organic, internally recycled phosphorus system as soils age and inorganic inputs diminish, consistent with other forest soil pedogenic gradients (Lang et al. 2016; Yu, Ahrens, Wutzler, Zaehle, and Schrumpf 2020).

Our simulations also highlight the increasing importance of living organisms as dynamic phosphorus reservoirs. In low‐phosphorus systems, microbial phosphorus exceeded plant stocks, underscoring their central role in nutrient retention and turnover. Field data in the Amazon and elsewhere show microbial phosphorus can comprise up to 68%–78% of total biological phosphorus in some forest soils (Fuchslueger et al. 2025; Turner et al. 2013). Rapid microbial turnover likely enhances phosphorus availability but may also intensify competition with plants, a symptom of nutrient stress in aging soils (Turner et al. 2013). Our simulations suggest plant biomass is also an important phosphorus reservoir (~5 g P m^−2^, ≈0.05 t P ha^−1^), though conservatively low compared to field estimates (0.2–0.9 t P ha^−1^; Bauters et al. 2022), indicating the plant reservoir is likely even larger.

### 4.3 Phosphorus Limitation on Carbon Sequestration

Increased CO_2_ stimulated primary production, litterfall, and heterotrophic respiration across all sites, accelerating carbon cycling. Although iCO_2_ enhanced belowground carbon allocation, it did not substantially increase soil carbon sequestration over the 119‐year simulation period. Most additional carbon was either stored temporarily in plant biomass or respired, with only modest accumulation in long‐lived soil carbon pools. This pattern aligns with findings from EucFACE, a phosphorus‐limited *Eucalyptus* forest, where increased belowground carbon allocation under iCO_2_ also failed to enhance soil carbon stocks (Jiang et al. 2020).

The average iCO_2_‐driven plant biomass increase was 2.9 kg C m^−2^, equivalent to 10% of extra photosynthetic carbon. Soil phosphorus modulated the magnitude of CO_2_ fertilization: plant biomass carbon accumulation was 40% lower in low‐phosphorus than high‐phosphorus sites. While covarying climate along the soil gradient prevents isolating pure phosphorus effects, the emergent differences in plant, soil and ecosystem phosphorus cycling can be attributed to soil phosphorus constraints. Low‐phosphorus sites were defined as those with total soil phosphorus < 80 g P m^−2^, based on the onset of nutrient limitation indicators in our simulation, though basin‐wide analyses suggest that field‐based thresholds for phosphorus limitations to tree growth and shifts in leaf N : P ratios may start around 60 g P m^−2^ (Fyllas et al. 2009; Quesada et al. 2010). Extrapolation from topsoil in situ measurements of phosphorus introduces uncertainty in the representation of deep soil phosphorus availability in the model, particularly as the current implementation does not explicitly resolve site‐specific soil phosphorus profiles or rooting depth distributions. Depending on the vertical distribution and accessibility of deep soil phosphorus, this simplification could lead to either under‐ or overestimation of progressive phosphorus limitation across sites. Future work should explore the integration of in situ soil phosphorus profiles and depth information (Quesada et al. 2011).

Understanding future phosphorus constraints on the tropical carbon sink remains challenging, as most Earth System Models still omit phosphorus cycling (Koch et al. 2021). Few phosphorus‐enabled terrestrial biosphere models are evaluated with Amazonian forest data. The model ELM‐CNP was parameterized with Amazonian forest plot data and captured productivity gradients across the basin (Yang et al. 2019) but lacked explicit microbial phosphorus cycling and did not integrate in situ soil data. Other models include demographic or nutrient feedbacks but their carbon‐phosphorus interactions under CO_2_ fertilization remain under‐evaluated (Knox et al. 2024; Nakhavali et al. 2022). Our study advances this field by integrating in situ soil and vegetation data, including belowground dynamics, highlighting the need for further observational constraints.

### 4.4 Organic Phosphorus Cycling

Our simulations indicate that iCO_2_ led to progressive phosphorus limitation by accelerating organic phosphorus turnover and depleting plant‐available pools. Plant phosphorus uptake increased across the entire gradient. Even in low‐phosphorus soils, plants required more phosphorus under iCO_2_ to support the rapid turnover of short‐lived tissues, benefiting from microbial‐driven mineralization of phosphorus. This response contrasts with observations suggesting that plants in nutrient‐poor environments conserve phosphorus through longer tissue lifespans and higher resorption (Lambers et al. 2008). Plant nutrient use efficiency and its upregulation under iCO_2_ may thus be more important than we have simulated. Nevertheless, increased carbon availability under iCO_2_ is expected to stimulate faster plant carbon turnover, yet to what degree that leads to heightened plant phosphorus demand and turnover remains uncertain.

Ecosystem response to this increased demand varied by available soil phosphorus forms. High‐phosphorus sites showed depletion of inorganic phosphorus pools, while low‐phosphorus sites increasingly relied on organic phosphorus, including mineral‐associated organic phosphorus, which were draw down under iCO_2_, signaling a shift toward more slowly replenished phosphorus sources. Investment in phosphatase‐driven biochemical mineralization rose disproportionately under iCO_2_, consistent with prior modeling studies (e.g., Fleischer et al. 2019; Yang et al. 2019) and global meta‐analyses (Margalef et al. 2021). These changes led to faster ecosystem phosphorus turnover and gradual depletion of soil organic phosphorus, emphasizing mineralization and recycling as key in meeting elevated phosphorus demands under CO_2_ enrichment (Sun et al. 2017). Also in temperate systems, higher microbial phosphorus uptake was symptomatic at the low end of the phosphorus gradient, which could be due to faster phosphorus turnover (Spohn et al. 2018). While increased phosphatase activity is often associated with higher mineralization potential, the consequence of net loss of soil organic phosphorus pools has rarely been explored in CO_2_ fertilization studies. Early field evidence in an Amazon Open Top Chamber experiment supports reductions in soil organic phosphorus under elevated CO_2_ (Martins et al. 2026).

Finally, iCO_2_ redistributed phosphorus from soil into plant and microbial biomass. While plant phosphorus increases align with observations, the simulated increase in microbial phosphorus is more uncertain, relying on model assumptions on stoichiometry and carbon use efficiency. For example, microbial phosphorus increase was not supported by field data at EucFACE (Jiang et al. 2024). Nonetheless, accelerated organic phosphorus turnover and rising competition between plants and microbes are robust signals of progressive phosphorus limitation under iCO_2_.

### 4.5 Model‐Based Hypotheses for CO_2_ Fertilization in Tropical Forests

Our model generates directly testable hypotheses for the ecosystem‐scale CO_2_ enrichment experiments in Brazil, AmazonFACE (Lapola et al. 2024). As previously suggested, increased fine root production and higher phosphate‐enzyme production under iCO_2_ are expected responses (Fleischer et al. 2019; Hofhansl et al. 2016; Yang et al. 2019). Here, we additionally identify signals of progressive phosphorus limitation: accelerated soil phosphorus cycling, depletion of mineral‐associated organic phosphorus pools, and potentially shorter plant carbon and phosphorus residence times. These responses emerge under low‐phosphorus conditions such as those found at the AmazonFACE site.

We hypothesize that in low‐phosphorus systems, the increased plant phosphorus demand under iCO_2_ is met primarily through enhanced organic phosphorus cycling. This would manifest as elevated enzyme activity and higher biochemical mineralization rates, particularly in the topsoil, where organic phosphorus is concentrated. The litter layer, although not explicitly represented in our model, is expected to contribute substantially to organic phosphorus supply in highly recycling systems (Herrera et al. 1978; Martins et al. 2021; Sayer et al. 2020; Schaap et al. 2021). Our simulations suggest that reliance on organic phosphorus sources constrains biomass responses to iCO_2_, a limitation likely to become even more pronounced over time. Alternative phosphorus acquisition pathways, such as organic acid exudation and mycorrhizal associations, are not yet included and may play additional roles under field conditions.

Our findings highlight that further model advances depend on improved empirical constraints. In particular, microbial enzyme production, stoichiometric flexibility, and (de)sorption and exchange kinetics remain underexplored in tropical low‐phosphorus systems (Allison et al. 2007; Helfenstein et al. 2018; Ma et al. 2023; Wang et al. 2022; Yu et al. 2023). At the same time, the contribution of additional plant phosphorus acquisition strategies, including root exudation, mycorrhizal associations, and their associated carbon costs, is still poorly quantified at ecosystem scale (Braghiere et al. 2022; Reichert et al. 2023; Schufft et al. 2026). Shift in plant nutrient acquisition strategies likely also shapes plant community composition, adding another layer of ecosystem response at play (Lambers et al. 2008; Reichert et al. 2022). Without better empirical constraints on these processes and their trade‐offs, increasing model complexity may simply amplify uncertainty. Coordinated model‐experiment efforts are therefore essential (Jiang et al. 2024). Experiments such as AmazonFACE provide a critical test whether the modeled acceleration of organic phosphorus cycling, shifts in nutrient acquisition strategies, and phosphorus limitation to plant carbon accumulation emerge under field conditions.

## 5 Conclusion

Our modeling study shows that under low‐phosphorus conditions, the organic phosphorus cycle plays a crucial role and may face greater pressure under rising CO_2_. Biochemical phosphorus mineralization emerges as a key mechanism sustaining productivity in a carbon‐rich world. Forests may increasingly rely on organic matter recycling, where phosphorus is continually transformed and reused rather than supplied by new mineral inputs. This points to a potential vulnerability of Amazon forests to the growing imbalance between CO_2_ concentrations and soil phosphorus limitation. At the same time, soil fertility drives different ecosystem responses, and the mosaic of soil conditions across the Amazon basin is likely to result in complex responses of forest carbon sequestration. Without alternative acquisition strategies, this recycling dependence could become a bottleneck for the carbon sink potential of the Amazon forests. We may also expect an increasing vulnerability of Amazon forests to disturbances or changes in nutrient inputs. We identify additional signatures of progressive phosphorus limitation under iCO_2_, including reduced soil organic phosphorus pools, accelerated phosphorus turnover, and potentially shorter residence times for plant carbon and phosphorus, parameters that need careful monitoring in field studies and experiments.

## Author Contributions

**Katrin Fleischer:** writing – original draft, writing – review and editing, visualization, formal analysis, methodology, conceptualization. **Carlos A. Quesada:** writing – review and editing. **Sönke Zaehle:** writing – review and editing, conceptualization, methodology. **Lucia Fuchslueger:** writing – review and editing, visualization. **Lin Yu:** methodology, conceptualization, writing – review and editing, formal analysis, visualization.

## Funding

K.F. acknowledges funding by the Max Planck Society, Max Planck Institute for Biogeochemistry (BGC‐Jena), and the Amsterdam Institute for Life and Environment (A‐LIFE) at the Vrije Universiteit Amsterdam. L.Y. acknowledges support from the CLICCS Cluster of Excellence funded by the DFG under Germany's Excellence Strategy (EXC 2037, Project No. 390683824). L.F. acknowledges the European Union's Horizon 2020 research and innovation program under the Marie Sklodovska‐Curie grant agreement No. 847693 (REWIRE). C.A.Q. acknowledges funding by the CNPq productivity grant (Process 312866/2021‐6).

## Conflicts of Interest

The authors declare no conflicts of interest.

## Supporting information


**Figure S1:** Top soil phosphorus gradient for Amazonian forest plots used in this study (*n* = 31), shown by (A) stacking mean phosphorus fractions in g P m^−2^ and (B) their relative fraction of total phosphorus in %, both aggregated per soil type in the order of presumed soil pedogenesis from youngest on the left to oldest on the right. Top soil phosphorus reflects total phosphorus in the top four soil layers in model simulations, equivalent to 26 cm soil depth.
**Figure S2:** Mean carbon and phosphorus cycling for Amazonian forest plots along the top soil phosphorus gradient (g P m^−2^) for the reference period 1999 to 2009, comparing model simulations (black) with field observations from forest census (gray) for plant wood carbon stock (g C m^−2^), word carbon loss from turnover (g C m^−2^ year^−1^), wood carbon residence time (years), soil carbon stock (g C m^−2^), soil nitrogen stock (g N m^−2^), leaf carbon : phosphorus ratio, and soil organic carbon : nitrogen ratio. Top soil phosphorus reflects total phosphorus in the top four soil layers in model simulations, equivalent to 26 cm soil depth.
**Figure S3:** Simulated mean carbon and phosphorus cycling for Amazonian forest plots along the top soil phosphorus gradient (g P m^−2^) for the reference period 1999–2009, including aboveground and belowground carbon allocation fraction (CG_above, CG_below; unitless), leaf area index (LAI; m^−2,^ m^−2^), leaf litterfall return of phosphorus (LeafLitterFall_P; g P m^−2^ year^−1^), microbial carbon use efficiency (MicrobialCUE, unitless), plant carbon use efficiency (PlantCUE; NPP/GPP, unitless), plant fine root carbon stock (PlantFRoot; g C m^−2^), plant phosphorus use efficiency (PlantPUE; unitless), plant biomass nitrogen : phosphorus ratio (PlantTotalNP; unitless), plant carbon residence time (PlantTotalResTime; years), litterfall return of carbon (TotalLitterFall_C; g C m^−2^ year^−1^), carbon : phosphorus ratio of total litterfall (TotalLitterFall_CP; unitless). Top soil phosphorus reflects total phosphorus in the top four soil layers in model simulations, equivalent to 26 cm soil depth.
**Figure S4:** Simulated CO_2_ effect on carbon cycling for the Amazonian forest plots after 119 years of increasing CO_2_ and climate change, shown as cross‐comparison of cumulative fluxes and soil carbon pool changes, including plant and soil carbon change (g C m^−2^), cumulative litterfall carbon, cumulative fine root litterfall carbon, and cumulative heterotrophic respiration (g C m^−2^).
**Figure S5:** Cumulative iCO_2_ effects on carbon and phosphorus fluxes for Amazonian forest plots along the top soil phosphorus gradient (g C/P m^−2^) after 119 years of increasing CO_2_ and climate change, including fine root carbon litter input (CFRLINsum), litter carbon input fraction of NPP (CLITT_NPPsum), total carbon litter input to soil (CLITT_SOILsum), gross primary productivity (GPPsum), net primary productivity (NPPsum), biochemical mineralization of phosphorus (PBIOMIN), gross mineralization of phosphorus (or depolymerization, PGMINsum), plant phosphorus uptake (PUPsum), phosphorus weathering (PWEAsum), fast exchange phosphorus input (PXFASTsum), slow exchange phosphorus input (PXSLOsum), heterotrophic respiration fraction of total carbon litter input to soil (RHET_CLITTsum), and heterotrophic respiration (RHETsum). Top soil phosphorus reflects total phosphorus in the top four soil layers in model simulations, equivalent to 26 cm soil depth.
**Figure S6:** Modeled plant carbon and phosphorus residence time in years for the Amazonian forest plots after the 119‐year simulation period, comparing the increasing and constant CO_2_ simulations. Residence time was calculated as total plant carbon or phosphorus divided by the sum of all litter fluxes from plant pools, representing the average time that carbon or phosphorus is retained in plant biomass before entering the litter pool.
**Figure S7:** Modeled iCO_2_ effects on soil phosphorus pools, shown as absolute change in g P m^−2^ for the Amazonian forest plots, after 119 years of increasing CO_2_ and climate change, including dissolved organic matter phosphorus in top and deep layer (PDOM_A/B), mineral‐associated dissolved organic matter phosphorus in top and deep layer (PaDOM_A/B), residue phosphorus (or necromass) in top and deep layer (PRES_A/B), mineral‐associated residue phosphorus (or necromass) in top and deep layer (PaRES_A/B), and microbial phosphorus in top and deep layer (PMICA/B).
**Table S1:** List of modeled Amazonian forest sites (*n* = 31) and their climate and soil phosphorus status, including their annual mean temperature, annual precipitation, top soil phosphorus (g P m^−2^), and soil phosphorus group, based on the respective climate input and modeled values averaged between 1991 and 2019. Sites were classified into the low soil phosphorus (< 80, *n* = 13), mid (> 80 and < 120, *n* = 8), and high soil phosphorus group (> 120, *n* = 10).
**Table S2:** Summary statistics and statistical comparisons for modeled iCO_2_ effects on plant, litter, soil, and microbial carbon and phosphorus pools and cumulative fluxes ((k)g C/P m^−2^) for Amazonian forest. Effects on pools are summarized over the last 3 years of the simulation, and cumulative fluxes are calculated after the entire simulation period. Values are shown as mean, SD, and median for forest sites grouped by low (*n* = 13) and high (*n* = 10) topsoil phosphorus content. Shapiro–Wilk tests were used to assess normality. Differences between groups were evaluated using two‐sample *t*‐tests or Wilcoxon rank‐sum tests depending on the normality assumption.
**Table S3:** Pairwise comparisons of temporal slopes among soil phosphorus groups. Slopes were estimated from linear mixed‐effects models of biotic phosphorus fraction (%) and ecosystem phosphorus turnover (%) over time, with centered year and soil phosphorus group as fixed effects and site as a random intercept. Reported values show the estimated difference in slopes between soil phosphorus groups (estimate), standard error (SE), and Tukey‐adjusted *p*‐values.

## Data Availability

Model simulation output and code are available under https://doi.org/10.5281/zenodo.20854682.
