# Supplementary material for: Simulated CO2 Fertilization Drives Progressive Phosphorus Limitation via Accelerated Organic Cycling in Amazonian Forests
Source: Glob Chang Biol. 2026 Jul 20;32(7):e71000. doi: 10.1111/gcb.71000 (PMC13382490; doi:10.1111/gcb.71000)
Supplement: Supplementary file 1 — Figure S1: Top soil phosphorus gradient for Amazonian forest plots used in this study (n = 31), shown by (A) stacking mean phosphorus fractions in g P m−2 and (B) their relative fraction of total phosphorus in %, both aggregated per soil type in the order of presumed soil pedogenesis from youngest on the left to oldest on the right. Top soil phosphorus reflects total phosphorus in the top four soil layers in model simulations, equivalent to 26 cm soil depth. Figure S2: Mean carbon and phosphorus cycling for Amazonian forest plots along the top soil phosphorus gradient (g P m−2) for the reference period 1999 to 2009, comparing model simulations (black) with field observations from forest census (gray) for plant wood carbon stock (g C m−2), word carbon loss from turnover (g C m−2 year−1), wood carbon residence time (years), soil carbon stock (g C m−2), soil nitrogen stock (g N m−2), leaf carbon : phosphorus ratio, and soil organic carbon : nitrogen ratio. Top soil phosphorus reflects total phosphorus in the top four soil layers in model simulations, equivalent to 26 cm soil depth. Figure S3: Simulated mean carbon and phosphorus cycling for Amazonian forest plots along the top soil phosphorus gradient (g P m−2) for the reference period 1999–2009, including aboveground and belowground carbon allocation fraction (CG_above, CG_below; unitless), leaf area index (LAI; m−2, m−2), leaf litterfall return of phosphorus (LeafLitterFall_P; g P m−2 year−1), microbial carbon use efficiency (MicrobialCUE, unitless), plant carbon use efficiency (PlantCUE; NPP/GPP, unitless), plant fine root carbon stock (PlantFRoot; g C m−2), plant phosphorus use efficiency (PlantPUE; unitless), plant biomass nitrogen : phosphorus ratio (PlantTotalNP; unitless), plant carbon residence time (PlantTotalResTime; years), litterfall return of carbon (TotalLitterFall_C; g C m−2 year−1), carbon : phosphorus ratio of total litterfall (TotalLitterFall_CP; unitless). Top soil phosphorus reflects total phosphor [file GCB-32-e71000-s001.docx]

**Table S1. List of modelled Amazonian forest sites (n=31) and their climate and soil phosphorus status**, including their annual mean temperature, annual precipitation, top soil phosphorus (g P m^-2^) and soil phosphorus group, based on the respective climate input and modelled values averaged between 1991 to 2019. Sites were classified into low soil phosphorus (< 80, n=13), mid (> 80 and < 120, n=8), and high soil phosphorus (>120, n=12).

| Site Code | Mean annual temperature | Annual precipitation | Soil phosphorus (top) | Soil phosphorus group |
| --- | --- | --- | --- | --- |
| ZAR-02 | 27.2 | 2615.9 | 40.6 | low |
| BNT-04 | 28.1 | 2256.4 | 45.6 | low |
| JEN-13 | 27.2 | 2729.3 | 48.8 | low |
| POR-02 | 26.6 | 2085.0 | 51.8 | low |
| ALF-01 | 27.2 | 2276.0 | 53.9 | low |
| CAX-06 | 27.9 | 2352.4 | 54.2 | low |
| JRI-01 | 27.2 | 2299.2 | 58.2 | low |
| SUC-02 | 27.1 | 3009.7 | 60.1 | low |
| CAX-02 | 27.9 | 2352.4 | 62.8 | low |
| BOG-02 | 25.9 | 3330.4 | 70.7 | low |
| MNU-03 | 25.9 | 3072.3 | 73.4 | low |
| LOR-01 | 27.0 | 2699.1 | 77.1 | low |
| TAM-07 | 26.0 | 2465.2 | 77.8 | low |
| BOG-01 | 25.9 | 3330.4 | 81.6 | mid |
| RST-01 | 26.7 | 2149.4 | 87.9 | mid |
| LOR-02 | 27.0 | 2699.1 | 97.6 | mid |
| TIP-03 | 25.9 | 3330.4 | 99.1 | mid |
| DOI-01 | 26.7 | 2050.6 | 103.3 | mid |
| AGP-02 | 27.0 | 2696.8 | 104.0 | mid |
| TAM-05 | 26.0 | 2465.2 | 112.0 | mid |
| AGP-01 | 27.0 | 2696.8 | 114.7 | mid |
| MNU-05 | 25.9 | 3072.3 | 132.1 | high |
| TAM-04 | 26.0 | 2465.2 | 132.9 | high |
| YAN-01 | 27.1 | 3009.7 | 135.1 | high |
| TAM-01 | 26.0 | 2465.2 | 137.0 | high |
| TAM-02 | 26.0 | 2465.2 | 144.4 | high |
| MNU-06 | 25.9 | 3072.3 | 165.4 | high |
| DOI-02 | 26.7 | 2050.6 | 165.5 | high |
| YAN-02 | 27.1 | 3009.7 | 180.2 | high |
| TAM-06 | 26.0 | 2465.2 | 214.8 | high |
| CUZ-03 | 26.2 | 2158.0 | 311.8 | high |

**Table S2. Summary statistics and statistical comparisons for modelled iCO₂ effects** on plant, litter, soil, and microbial carbon and phosphorus pools and cumulative fluxes ((k)g C/P m⁻²) for Amazonian forest. Effects on pools are summarized over the last 3 years of the simulation, and cumulative fluxes are calculated after the entire simulation period. Values are shown as mean, SD, and median for forest sites grouped by low (n = 13) and high (n = 10) topsoil phosphorus content. Shapiro–Wilk tests were used to assess normality. Differences between groups were evaluated using two-sample t-tests or Wilcoxon rank-sum tests depending on the normality assumption.

|  | Mean  low P | Mean  high P | SD  low P | SD  high P | Median  low P | Median  high P | Shapiro p  low P | Shapiro p  high P | t-test p | Wilcox p | Test Used |
| --- | --- | --- | --- | --- | --- | --- | --- | --- | --- | --- | --- |
| **Pools** |  |  |  |  |  |  |  |  |  |  |  |
| Plant C | 2208.1 | 3690 | 371.9 | 537.7 | 2205.9 | 3696.1 | 0.568 | 0.174 | <0.001 | <0.001 | t-test |
| Microbial C | 46 | 35.4 | 6.7 | 8.6 | 47.6 | 33.6 | 0.09 | 0.59 | 0.005 | 0.01 | t-test |
| Fine root C | 55.4 | 25 | 10.6 | 12.5 | 56.7 | 18.7 | 0.786 | 0.005 | <0.001 | <0.001 | Wilcoxon |
| Soil C | 445.2 | 410.6 | 104.4 | 79.5 | 448.6 | 424.8 | 0.478 | 0.284 | 0.376 | 0.376 | t-test |
| Plant P | 0.6 | 0.9 | 0.1 | 0.2 | 0.6 | 1 | 0.179 | 0.308 | 0.002 | <0.001 | t-test |
| Microbial P | 1.1 | 0.8 | 0.2 | 0.2 | 1.1 | 0.8 | 0.09 | 0.59 | 0.005 | 0.01 | t-test |
| Soil inorg P | -0.2 | -4.2 | 0.3 | 2.5 | 0.1 | -4.6 | 0.013 | 0.051 | <0.001 | <0.001 | Wilcoxon |
| Soil MA-org P | -2.4 | -0.1 | 0.6 | 1.9 | -2.7 | 0.4 | 0.677 | 0.004 | 0.004 | 0.012 | Wilcoxon |
|  |  |  |  |  |  |  |  |  |  |  |  |
| **Cumulative Fluxes** |  |  |  |  |  |  |  |  |  |  |  |
| GPP | 28208.6 | 31845.2 | 1828.8 | 1417.8 | 27643 | 32102.4 | 0.288 | 0.115 | <0.001 | <0.001 | t-test |
| NPP | 9091.7 | 10631.7 | 682.1 | 829.7 | 9262.9 | 10794.5 | 0.375 | 0.032 | <0.001 | <0.001 | Wilcoxon |
| Fine root litterfall | 3052.4 | 1361.2 | 659.6 | 739.6 | 2940.7 | 1027.3 | 0.486 | <0.001 | <0.001 | <0.001 | Wilcoxon |
| Het. Respiration | 6195 | 6255.1 | 585 | 344.5 | 6365.9 | 6353.1 | 0.027 | 0.006 | 0.762 | 0.927 | Wilcoxon |
| Biochem. P min. | 69.3 | 40.1 | 10.1 | 15.2 | 71.9 | 33.8 | 0.268 | 0.002 | <0.001 | <0.001 | Wilcoxon |
| P gross min. | 6.5 | 7.3 | 1.7 | 1.6 | 6.6 | 7.1 | 0.082 | 0.048 | 0.307 | 0.313 | Wilcoxon |
| Total P min. | 75.8 | 47.4 | 9.7 | 15.3 | 77.7 | 40.3 | 0.611 | 0.004 | <0.001 | <0.001 | Wilcoxon |
| P desorption | 0.1 | 3.7 | 0.2 | 2.2 | 0 | 4 | 0.105 | 0.047 | <0.001 | 0.001 | Wilcoxon |

**Table S3. Pairwise comparisons of temporal slopes among soil phosphorus groups.** Slopes were estimated from linear mixed-effects models of biotic phosphorus fraction (%) and ecosystem phosphorus turnover (%) over time, with centered year and soil P group as fixed effects and site as a random intercept. Reported values show the estimated difference in slopes between soil P classes (estimate), standard error (SE), and Tukey-adjusted p-values.

| Comparison | Estimate | SE | p-value |
| --- | --- | --- | --- |
| **Biota P** |  |  |  |
| 0–80 - 80–120 | 0.010235 | 0.000844 | <0.001 |
| 0–80 - 120–200 | 0.014856 | 0.000844 | <0.001 |
| 0–80 - >200 | 0.01963 | 0.001427 | <0.001 |
| 80–120 - 120–200 | 0.004621 | 0.000939 | <0.001 |
| 80–120 - >200 | 0.009395 | 0.001485 | <0.001 |
| 120–200 - >200 | 0.004775 | 0.001485 | 0.007 |
|  |  |  |  |
| **P turnover** |  |  |  |
| 0–80 - 80–120 | 0.021253 | 0.001257 | <0.001 |
| 0–80 - 120–200 | 0.028083 | 0.001257 | <0.001 |
| 0–80 - >200 | 0.038389 | 0.002125 | <0.001 |
| 80–120 - 120–200 | 0.00683 | 0.001399 | <0.001 |
| 80–120 - >200 | 0.017135 | 0.002212 | <0.001 |
| 120–200 - >200 | 0.010306 | 0.002212 | <0.001 |

**
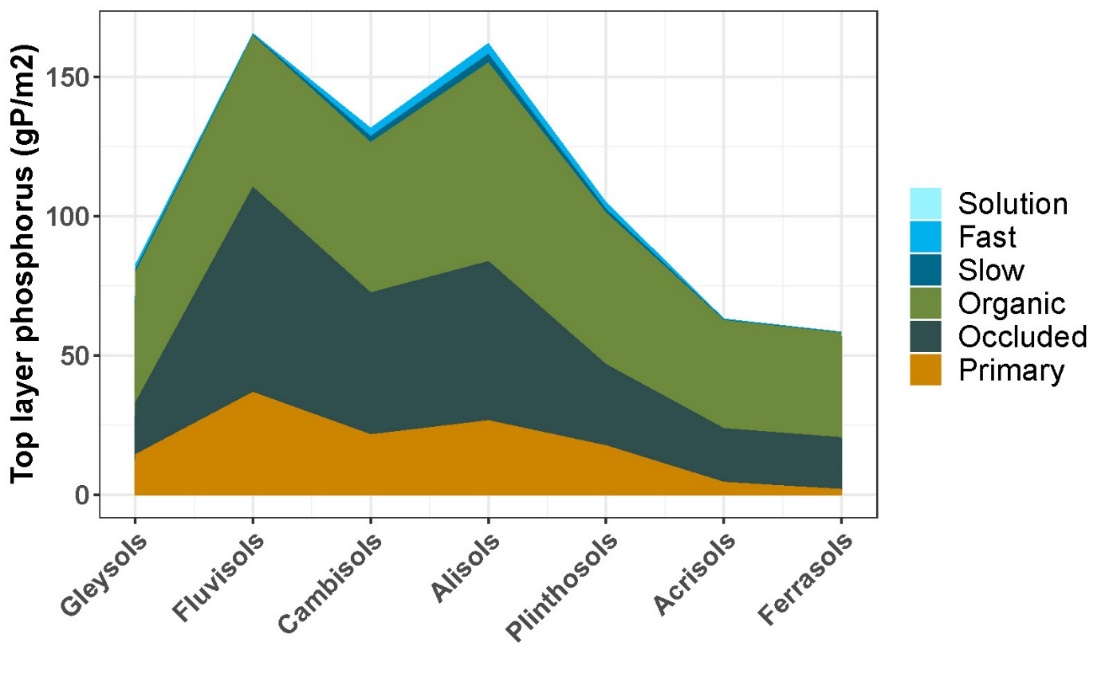

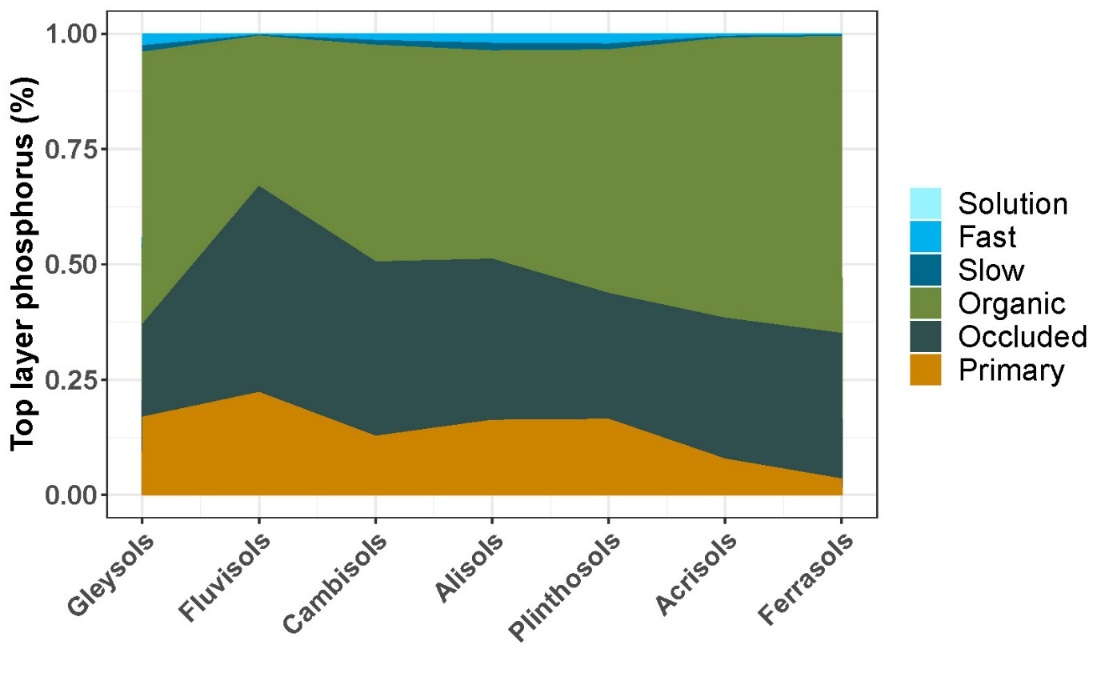
**

**Figure S1. Top soil phosphorus gradient for Amazonian forest plots used in this study (n=31)**, shown by (A) stacking mean phosphorus fractions in g P m^-2^ and (B) their relative fraction of total phosphorus in %, both aggregated per soil type in the order of presumed soil pedogenesis from youngest on the left to oldest on the right. Top soil phosphorus reflects total phosphorus in the top four soil layers in model simulations, equivalent to 26 cm soil depth.


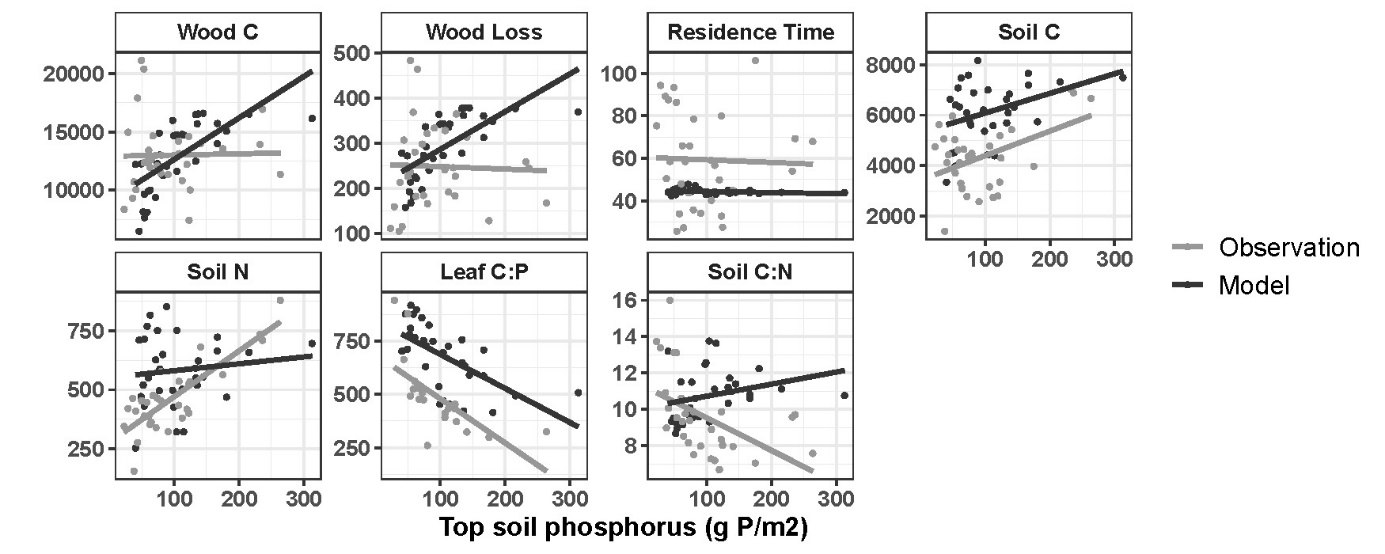


**Figure S2. Mean carbon and phosphorus cycling for Amazonian forest plots along the top soil phosphorus gradient (g P m^-2^) for the reference period 1999 to 2009**, comparing model simulations (black) with field observations from forest census (grey) for plant wood carbon stock (g C m^-2^), word carbon loss from turnover (g C m^-2^ yr^-1^), wood carbon residence time (years), soil carbon stock (g C m^-2^), soil nitrogen stock (g N m^-2^), leaf carbon:phosphorus ratio, and soil organic carbon:nitrogen ratio. Top soil phosphorus reflects total phosphorus in the top four soil layers in model simulations, equivalent to 26 cm soil depth.


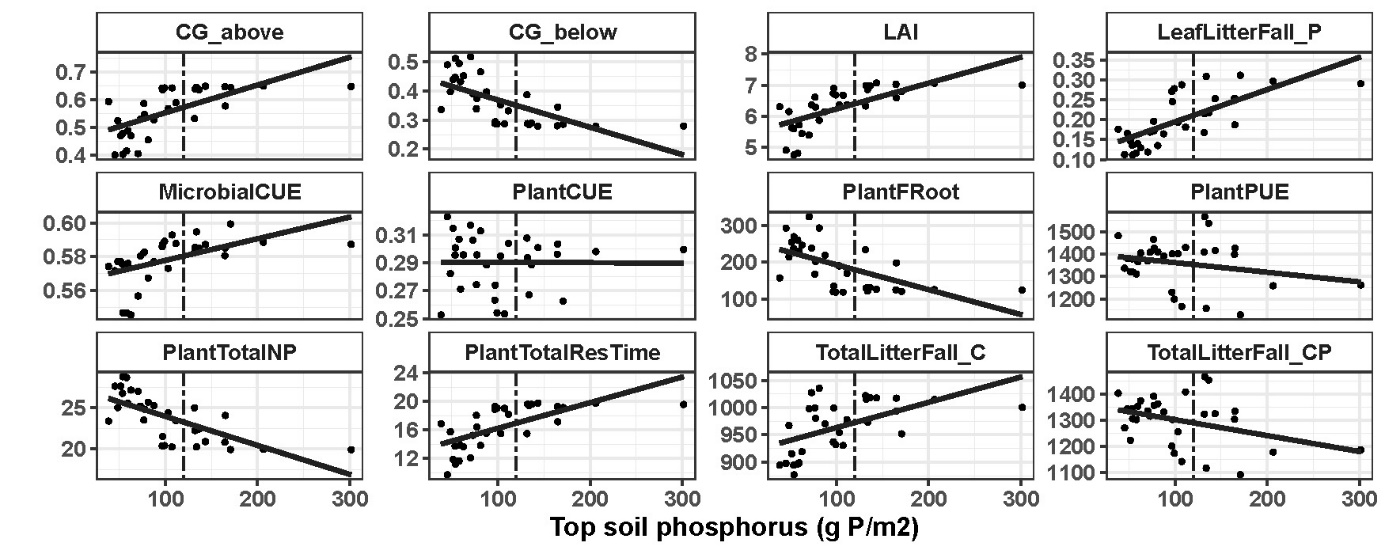


**Figure S3. Simulated mean carbon and phosphorus cycling for Amazonian forest plots along the top soil phosphorus gradient (g P m^-2^) for the reference period 1999 to 2009**, including aboveground and belowground carbon allocation fraction (CG_above, CG_below; unitless), leaf area index (LAI; m^-2,^ m^-2^), leaf litterfall return of phosphorus (LeafLitterFall_P; g P m^-2^ yr^-1^), microbial carbon use efficiency (MicrobialCUE, unitless), plant carbon use efficiency (PlantCUE; NPP/GPP, unitless), plant fine root carbon stock (PlantFRoot; g C m^-2^), plant phosphorus use efficiency (PlantPUE; unitless), plant biomass nitrogen:phosphorus ratio (PlantTotalNP; unitless), plant carbon residence time (PlantTotalResTime; years), litterfall return of carbon (TotalLitterFall_C; g C m^-2^ yr^-1^), carbon:phosphorus ratio of total litterfall (TotalLitterFall_CP; unitless). Top soil phosphorus reflects total phosphorus in the top four soil layers in model simulations, equivalent to 26 cm soil depth.


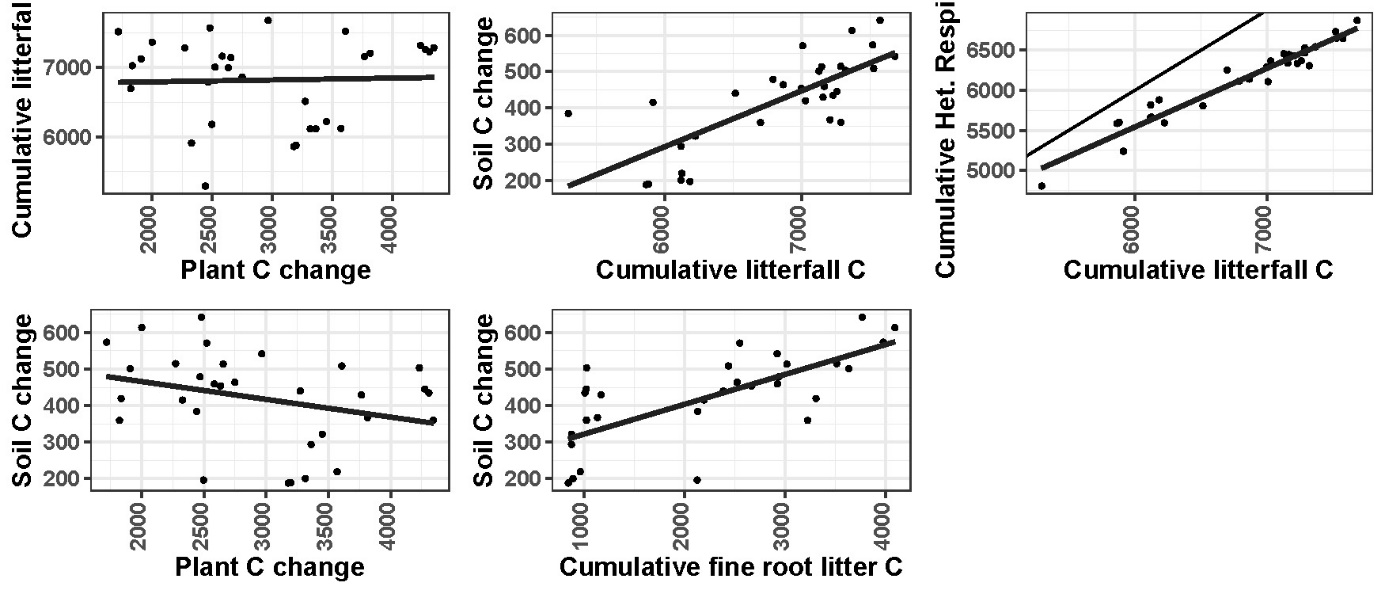


**Figure S4. Simulated CO_2_ effect on carbon cycling for the Amazonian forest plots** after 119 years of increasing CO_2_ and climate change, shown as cross-comparison of cumulative fluxes and soil carbon pool changes, including plant and soil carbon change (g C m^-2^), cumulative litterfall carbon, cumulative fine root litterfall carbon, and cumulative heterotrophic respiration (g C m^-2^).


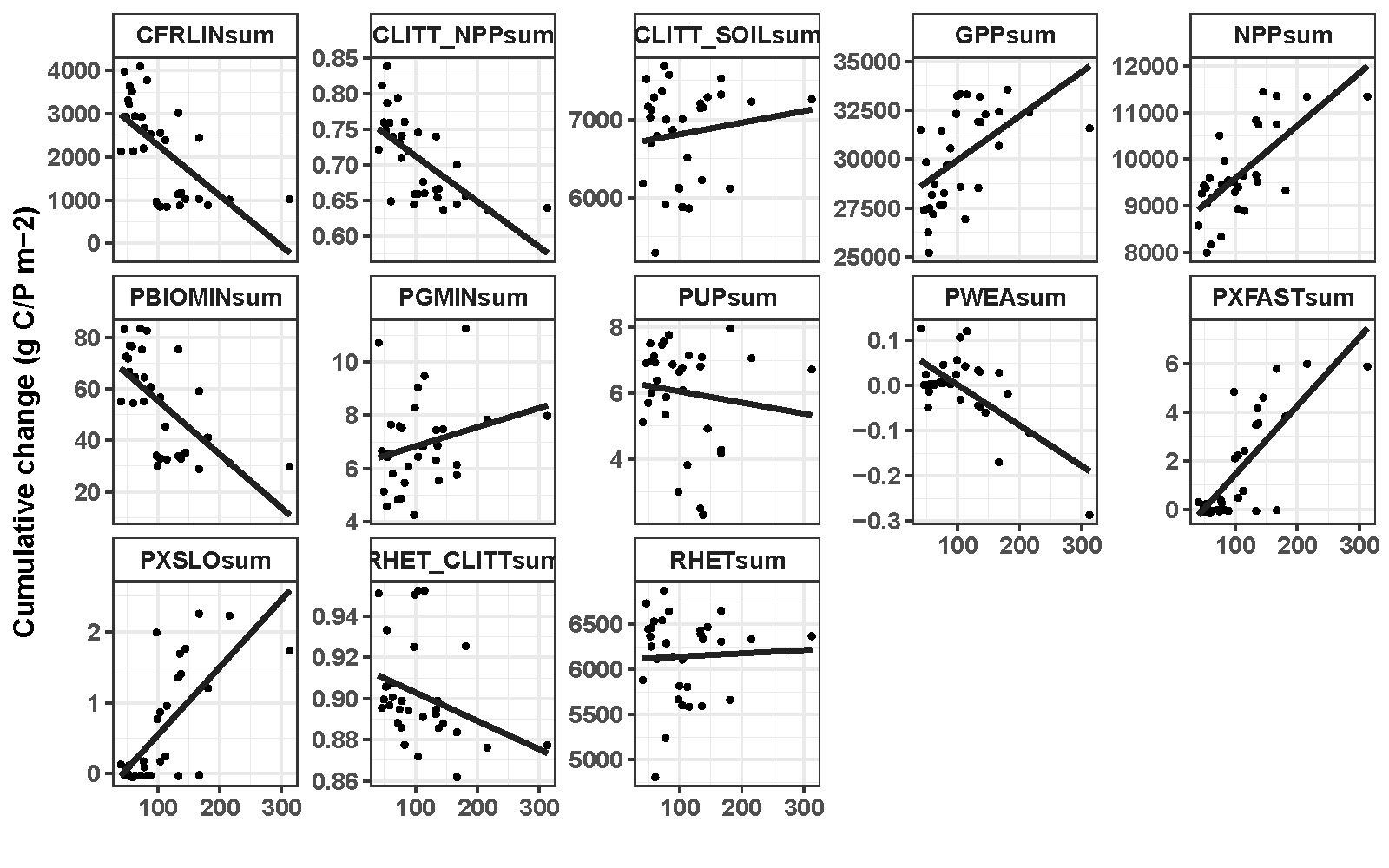


**Figure S5. Cumulative iCO_2_ effects on carbon and phosphorus fluxes for Amazonian forest plots along the top soil phosphorus gradient (**g C/P m^-2^ **)** after 119 years of increasing CO_2_ and climate change, including fine root carbon litter input (CFRLINsum), litter carbon input fraction of NPP (CLITT_NPPsum), total carbon litter input to soil (CLITT_SOILsum), gross primary productivity (GPPsum), net primary productivity (NPPsum), biochemical mineralization of phosphorus (PBIOMIN), gross mineralization of phosphorus (or depolymerization, PGMINsum), plant phosphorus uptake (PUPsum), phosphorus weathering (PWEAsum), fast exchange phosphorus input (PXFASTsum), slow exchange phosphorus input (PXSLOsum), heterotrophic respiration fraction of total carbon litter input to soil (RHET_CLITTsum), and heterotrophic respiration (RHETsum). Top soil phosphorus reflects total phosphorus in the top four soil layers in model simulations, equivalent to 26 cm soil depth.


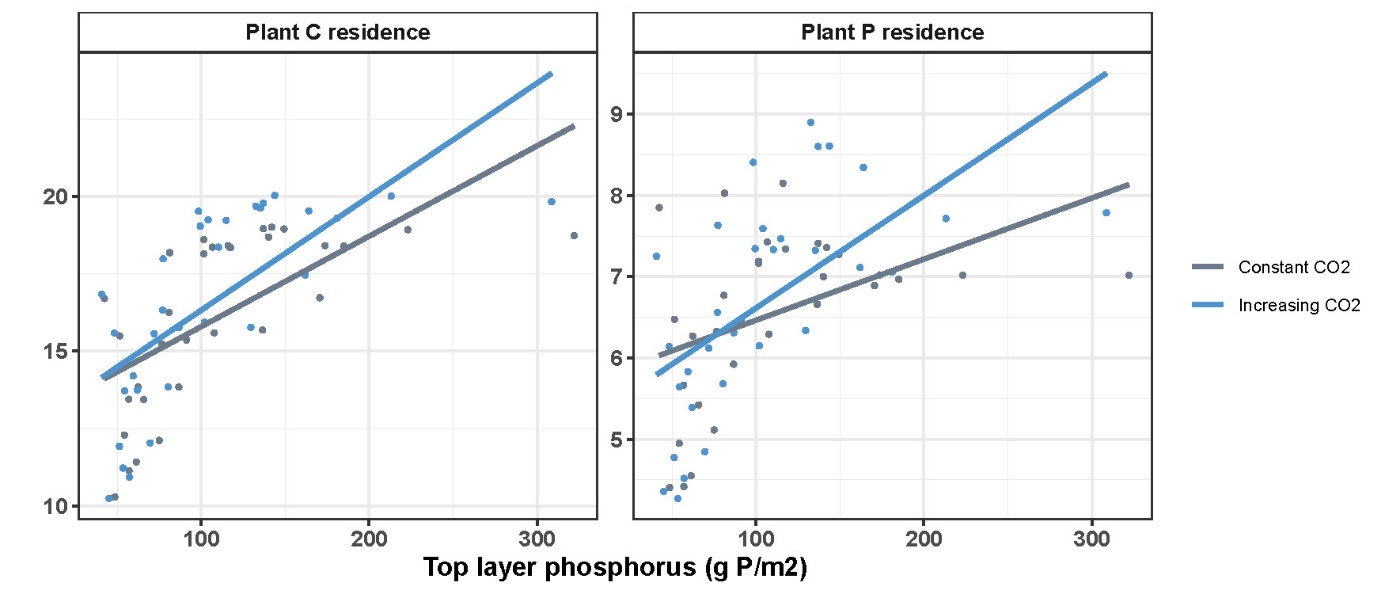


**Figure S6. Modelled plant carbon and phosphorus residence time** in years for the Amazonian forest plots after the 119-year simulation period, comparing the increasing and constant CO_2_ simulations. Residence time was calculated as total plant carbon or phosphorus divided by the sum of all litter fluxes from plant pools, representing the average time that carbon or phosphorus is retained in plant biomass before entering the litter pool.


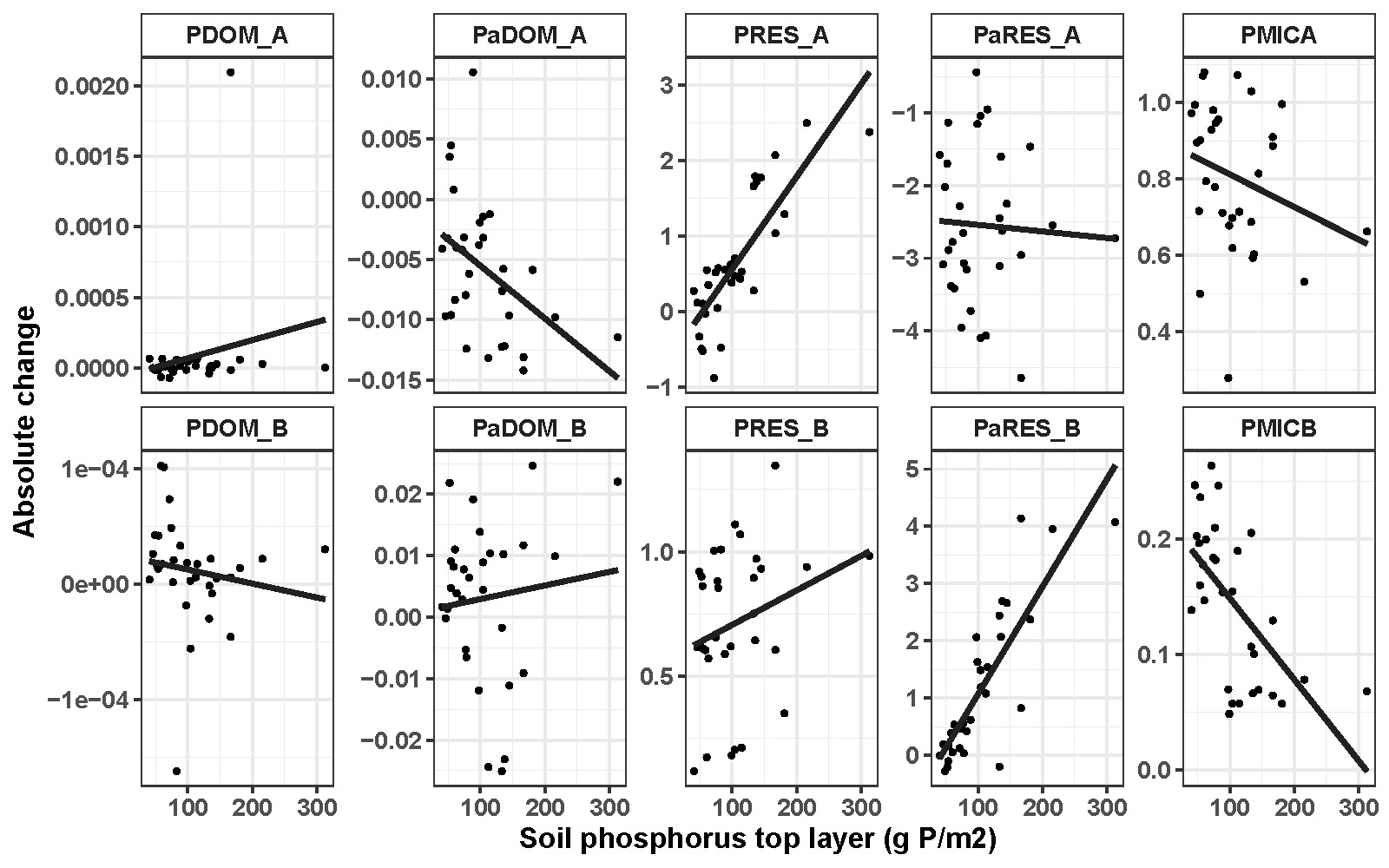


**Figure S7. Modelled iCO_2_ effects on soil phosphorus pools,** shown as absolute change in g P m^-2^ for the Amazonian forest plots, after 119 years of increasing CO_2_ and climate change, including dissolved organic matter phosphorus in top and deep layer (PDOM_A/B), mineral-associated dissolved organic matter phosphorus in top and deep layer (PaDOM_A/B), residue phosphorus (or necromass) in top and deep layer (PRES_A/B), mineral-associated residue phosphorus (or necromass) in top and deep layer (PaRES_A/B), and microbial phosphorus in top and deep layer (PMICA/B).
